# Supplementary material for: 3000 yr-old patterns of mobile pastoralism revealed by multiple isotopes and radiocarbon dating of ancient horses from the Mongolian Altai
Source: PLoS One. 2025 May 7;20(5):e0322431. doi: 10.1371/journal.pone.0322431 (PMC12057990; doi:10.1371/journal.pone.0322431)
Supplement: S1 File — (DOCX) [file pone.0322431.s001.docx]

**Supplementary material** **SM1-SM6**

**3000 yr-old patterns of mobile pastoralism revealed by multiple isotopes and radiocarbon dating of ancient horses from the Mongolian Altai.**

Antoine Zazzo^1*^, Maël Le Corre^1,2^, Nicolas Lazzerini^1^, Charlotte Marchina^3,4^, Noost Bayarkhuu^5,6^, Vincent Bernard^7^, Mathilde Cervel^8^, Denis Fiorillo^1^, Dominique Joly^9^, Michel Lemoine^1^, Philippe Telouk², François Thil^10^, Tsagaan Turbat^5^, Vincent Balter^2^, Aurélie Coulon ^11, 12^, Sébastien Lepetz^1^

^1^ Bioarchéologie, Interactions Sociétés Environnements (BioArch, UMR 7209), Muséum National d’Histoire Naturelle, Sorbonne Université, Centre National de la Recherche Scientifique (CNRS), CP 56, 55 rue Buffon, 75005 Paris, France.

^2^ Laboratoire de Géologie de Lyon, Terre, Planètes, Environnement (LGLTPE, UMR 5276), École Normale Supérieure Lyon, Université Lyon 1, Centre National de la Recherche Scientifique (CNRS), 46 Allée d’Italie, 69342 Lyon Cedex 07, France.

^3^ Institut Français de Recherche sur l’Asie de l’Est (IFRAE, UMR 8043), Institut National des Langues et Civilisations Orientales (Inalco), Université de Paris, Centre National de la Recherche Scientifique (CNRS), 2 Rue de Lille, 75007 Paris, France.

^4^ Institut universitaire de France (IUF), 1 rue Descartes, 75231 Paris, France.

^5^ Institute of Nomadic Archaeology and Department of Anthropology and Archaeology, National University of Mongolia, Ikh Surguuli Street 1, 14200 Ulaanbaatar, Mongolia.

^6^ Leibniz-Zentrum für Archäologie, Ludwig-Lindenschmit-Forum-1, 55116 Mainz, Germany.

^7^ Centre de Recherche en Archéologie, Archéosciences, Histoire (CReAAH, UMR 6566), Université Rennes 1, Centre National de la Recherche Scientifique (CNRS), Campus de Beaulieu, bat. 24/25, 35042 Rennes, France.

^8^ Archéologie & Philologie d’Orient et d’Occident (OOROC, UMR 8546), Université Paris Sciences & Lettres, Centre National de la Recherche Scientifique (CNRS), ENS-EPHE, 45 rue d’Ulm, 75230 Paris, France.

^9^ 3 quai de la Gloriette, 28000 Chartres, France.

^10^ Laboratoire des Sciences du Climat et de l’Environnement (LSCE/IPSL, UMR 8212), Commissariat à l'énergie atomique et aux énergies alternatives (CEA), Centre National de la Recherche Scientifique (CNRS), Université de Versailles – Saint-Quentin-en-Yvelines (UVSQ), 91198 Gif-sur-Yvette, France.

^11^ Centre d’Écologie et des Sciences de la Conservation (CESCO), Muséum National d’Histoire Naturelle, Centre National de la Recherche Scientifique (CNRS), Sorbonne Université, CP 135, 57 rue Cuvier, 75005 Paris, France

^12^ Centre d’Ecologie Fonctionnelle et Evolutive (CEFE), Centre National de la Recherche Scientifique (CNRS), École Pratique des Hautes Études (EPHE), Institut de Recherche pour le Développement (IRD), Université Paul Valéry Montpellier 3, 34090 Montpellier, France.

*corresponding author: [antoine.zazzo@mnhn.fr](mailto:antoine.zazzo@mnhn.fr)

**Supplementary material SM1. Horses’ information.**

**Table S1. Biological information of the horses from the Burgast site and the modern horse from the Mongolian Altai.** LBA = Late Bronze Age.

| **individual** | **estimated age at death** | **sex** | **Period** | **teeth sampled** |
| --- | --- | --- | --- | --- |
| ST61 | 1 | unknown | LBA | M_1_, M_2_ right |
| ST62 | 18-20 | female | LBA | M_1_, M_2_, M_3_ right |
| ST63 | adult (young) | unknown | LBA | M_1_, M_2_, M_3_ left |
| ST65A | young | male | LBA | M^1^, M^2^, M^3^ right |
| ST65B | very young | unknown | LBA | M_1_, M_2_, M_3_ left |
| ST66 | 5 | male | LBA | M^1^, M^2^, M^3^ right |
| ST67 | adult | unknown | LBA | M_1_, M_2_, M_3_ left |
| 2017-104 | 3.5 | female | modern | M_2_ |

**Supplementary material SM2. Mass spectrometry.**

**Table S2. LA-MC-ICP-MS operating parameters**

| **Parameters** | **Values** |
| --- | --- |
| *Laser ablation 193 nm ESL NWR* |  |
| Wavelength | 193 nm |
| He flow rate | 0.8 L/min |
|  |  |
| *Ablation* |  |
| Spot size | 100 μm |
| Frequency | 10 Hz |
| Fluence | 11 J/cm² |
| Sampling scheme | Line (dynamic) |
| Line translation rate | 60 μm/s |
| sample Line length | Depending on tooth |
| standard line length | 3 mm |
|  |  |
| *MC-ICP-MS Thermo-Fisher Neptune Plus^TM^* |  |
| Argon cool gas flow rate | 15 L/min |
| Auxillary gas flow | 1.0 L/min |
| Sample gas flow | 0.7 L/min |
| Plasma power resolution | 1200 |
|  |  |
| *Data collection* |  |
| Gas background | 30 s |
| Sample | Depending on tooth |
| Integration | 0.131 s |

**Supplementary material SM3. Radiocarbon dating.**

**Table S3. Radiocarbon age of the Late Bronze Age horses and human from the Burgast khirgisuur**

| individual | Species | ECHo # | Collagen | C:N ratio | ^14^C age | error | Calibrated range (95.4%) | |
| --- | --- | --- | --- | --- | --- | --- | --- | --- |
|  |  |  | yield (%) |  |  |  | from | to |
| 60 | Human | 1506 | 10.6 | 3.21 | 2870 | 25 | -1125 | -931 |
| 61 | Horse | 1507 | 9.1 | 3.27 | 2815 | 30 | -1054 | -854 |
| 62 | Horse | 1508 | 17.5 | 3.27 | 2860 | 25 | -1117 | -931 |
| 63 | Horse | 1509 | 12.2 | 3.27 | 2840 | 25 | -1108 | -917 |
| 64 | Horse | 1510 | 19.1 | 3.25 | 2850 | 25 | -1110 | -927 |
| 65A | Horse | 1511 | 14.5 | 3.24 | 2875 | 25 | -1188 | -934 |
| 65B | Horse | 1512 | 5.0 | 3.22 | 2845 | 30 | -1111 | -921 |
| 66 | Horse | 1513 | 15.3 | 3.27 | 2885 | 25 | -1196 | -940 |
| 67 | Horse | 1514 | 10.9 | 3.27 | 2875 | 30 | -1193 | -931 |

**Supplementary material SM4. Bioavailable ^87^Sr/^86^Sr isoscape modelling.**

We generated a bioavailable ^87^Sr/^86^Sr isoscape for Mongolia, updating an already existing global bioavailable ^87^Sr/^86^Sr isoscape from Bataille et al. [1] with local ^87^Sr/^86^Sr data from plants. To build their global bioavailable ^87^Sr/^86^Sr isoscape, Bataille et al. [1] used a random forest regression (RF), a machine-learning algorithm, to predict the spatial distribution of ^87^Sr/^86^Sr at very large scale, across the world. However, most of the more than 4000 sampling sites used to build the isoscape are located in North America and Europe with very few sampling locations from Asia and none from Mongolia. In such poorly informed regions, the isoscape shows a low prediction accuracy [1,2] and requires adding samples specific to these areas to improve local and regional predictions [1,2]. We used an updated version of the Bataille et al. [1] bioavailable ^87^Sr/^86^Sr dataset [2], and added recently published 156 local ^87^Sr/^86^Sr plant data from Altai [3], as well as plant and soil data from Asia listed in Table S8. We filtered the database and selected data from plants, soil and animals with limited movement range to ensure local ^87^Sr/^86^Sr values.

To predict the spatial distribution of the bioavailable ^87^Sr/^86^Sr, the RF integrates lithological, environmental and climate variables observed at each sampling site that likely influence the ^87^Sr/^86^Sr value in the environment [1]. We used the same set of variables, in raster format, as in Le Corre et al. [2] including minimal/maximal age and predicted ^87^Sr/^86^Sr value of the bedrock [4], terrane age, topography, soil properties, climate and salt and dust deposits. The list and details of all the predictors are provided in Table S4. Using R v4.3 [5], we extracted these variables at each ^87^Sr/^86^Sr sampling site. When a variable was not available at a given site due to lower raster resolution, accuracy or coverage, we used the nearest value in the vicinity of the site instead. RF was then applied to the ^87^Sr/^86^Sr sampling site dataset and the associated covariates, using soil, plant and animal ^87^Sr/^86^Sr values [1,2].

RF grows multiple regression trees by bagging [6]: for each tree, the dataset is divided by bootstrap into a training dataset and a validation dataset or “out-of-bag”. A regression tree is grown on the training dataset using a subset of predictors selected randomly, and the validation dataset is used for internal cross-validation. The outcome of each tree is then aggregated to obtain the predictions. RF does not make assumptions on data distribution and homoscedasticity, allowing handling complex relationships in the data [6]. We conducted our analysis in R v4.3 [5], following the workflow described in Le Corre et al. [2]. As a first step, we removed highly correlated variables (R > 0.9, median bedrock model and mean geological age variables) and applied a variable selection algorithm designed for RF (*VSURF* R package [7]) to remove irrelevant and redundant predictors. The remaining covariates were then used to run the final RF model. We set the number of trees to be grown to 3000 and the number of covariates used to grow the trees to the total number of covariates divided by three [6]. Root mean squared error (RMSE) and ten-fold cross-validation (repeated 5 time) were used to evaluate model performance and node impurity (i.e. a measure of the efficiency of the trees to split the training dataset in two groups at each node) was used to assess variable importance. The model was then applied to covariate rasters to predict ^87^Sr/^86^Sr across Mongolia. Finally, we used a quantile random forest regression to generate a 68,27% prediction interval and estimate the spatial uncertainty (standard deviation) associated with the prediction [8].

The global ^87^Sr/^86^Sr samples database (Table S8) used to train the random forest regression to generate the bioavailable ^87^Sr/^86^Sr isoscape for Mongolia is available as a separated Excel spreadsheet.

**Table S4. Auxiliary variables used in the random forest analysis.** The bedrock model [1] estimates the distribution of predicted ^87^Sr/^86^Sr values within lithological units based on their age and type [4]. GLiM: Global Lithological Map.

| Variables | Description | Resolution | References |
| --- | --- | --- | --- |
| r.m1 | median bedrock model | 1 km | [1] |
| r.srsrq1 | 1st quartile bedrock model | 1 km | [1] |
| r.srsrq3 | 3rd quartile bedrock model | 1 km | [1] |
| r.meanage_geol | mean GLiM age (Myrs) | 1 km | [9] |
| r.minage_geol | minimal GLiM age (Myrs) | 1 km | [9] |
| r.maxage_geol | maximal GLiM age (Myrs) | 1 km | [9] |
| r.age | terrane age (Myrs) | 1 km | [10] |
| r.mat | mean annual temperature (°C) | 30-arc sec | [11] |
| r.map | mean annual precipitation (mm.yrs^-1^) | 30-arc sec | [11] |
| r.pet | global potential evapo-transpiration | 30-arc sec | [12] |
| r.ai | global aridity index | 30-arc sec | [12] |
| r.salt | Simulation of sea salt deposition (g.m^-2^.yr^-1^) | 1° x 1° | [13] |
| r.dust | dust deposition (g.m^-2^.yr^-1^) | 1° x 1° | [14] |
| r.elevation | shuttle radar topography mission (m) | 90m | [15] |
| r.bouguer | bouguer anomaly | 2 min | [16] |
| r.GUM | global unconsolidated sediment map | 1 km | [17] |
| r.cec | cation exchange capacity (mmol(c)/kg) | 250 m | [18] |
| r.ph | soil pH (H2O, x10) | 250 m | [18] |
| r.phkcl | soil pH (KCl, x10) | 250 m | [18] |
| r.clay | clay (g/kg) | 250 m | [18] |
| r.ocs | organic carbon stocks (t/ha) | 250 m | [18] |
| r.bulk | bulk density (cg.cm-3) | 250 m | [18] |

**
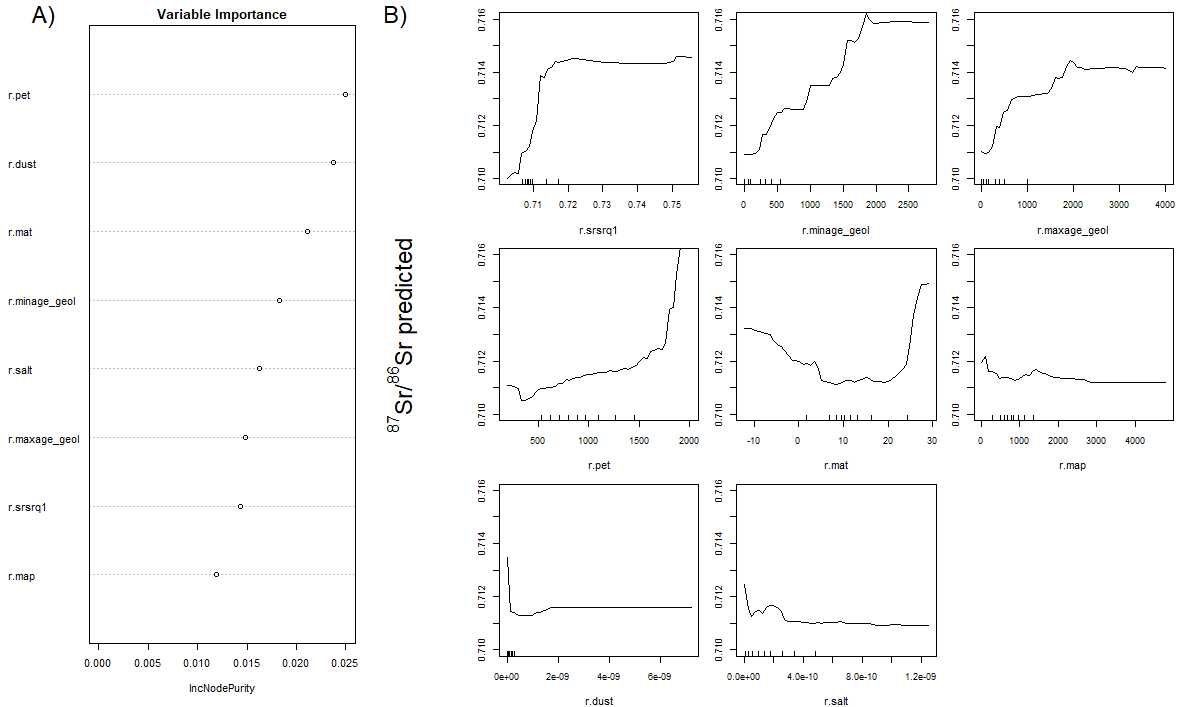
**

**Fig S1. Variable importance and partial dependence plots of the random forest regression.** A) Variable importance plot depicting the influence of the different predictors on the predicted bioavailable ^87^Sr/^86^Sr. B) Partial dependence plots depicting the relationship between the predictors retained for the final model and the predicted bioavailable ^87^Sr/^86^Sr. Hash marks on the x-axes correspond to the deciles of the predictor distribution. Description of the predictors are provided in Table S3.

**Supplementary material SM5. Descriptive statistics and inter-individual comparison for δ^18^O, δ^13^C and ^87^Sr/^86^Sr**

For δ^18^O, mean values between individuals vary significantly (Anova: *F*_7,430_ = 26.12, p-value < 0.001). On average, individuals have a δ^18^O value of -12.8 ‰ (± 2.13 SD). For all Bronze age horses but ST61, δ^18^O ranges from -18.0 ‰ to -9.3 ‰ (Table S6). Post-hoc Tukey test indicates that ST61 presents significantly higher δ^18^O values compared to the others (Fig S4A), notably on its M_2_ ranging from -9.2 ‰ to -5.7 ‰, while ST63 presents the significantly lowest δ^18^O values (Figure S4A) with the average δ^18^O values of its 3 molars below -14 ‰ (Table S6). The other individuals, including the modern 2017-104, show similar δ^18^O values (Figure S4A). Amplitude in δ^18^O is on average 4.9 ‰ (± 1.49).

For δ^13^C, individuals have an average value of -12.7 ‰ (± 0.63, Table S6). δ^13^C values range from -15.4 ‰ to -10.7 ‰ and are significantly different among individuals (*F*_7,430_ = 49.32, p-value < 0.001). It is noteworthy that ST61 presents significantly lower values than the other horses (Fig S4B), notably on its M_2_ ranging from -13.4 ‰ to -12.6 ‰ than the other horses. On the other hand, ST65a, ST65b, ST66 and ST67 present similar δ^13^C values, significantly higher than for the other horses (Fig S4B). Average amplitude is 1.4 ‰ (± 0.50) and varies from 0.4 ‰ to 2.6 ‰. Intra-tooth profiles vary between individuals without strong patterns (Fig 4). However, an increase in δ^13^C value along the teeth can be observed for horses ST63, ST65a, ST66 and ST67. Horses ST65b and, to a lesser extent, ST66, show stable values between -11 ‰ and -10 ‰. Only ST65a-M_2_, ST65b-M_1_ and ST66-M^1^ present a significant negative correlation between δ^18^O and δ^13^C values (Table S6)

Enamel ^87^Sr/^86^Sr ratios vary between 0.7051 and 0.7150 with an average of 0.7119 (± 0.0016). Amplitude in the signal is on average 0.0034 (± 0.0015), ranging from 0.0013 to 0.0069. ^87^Sr/^86^Sr varies between individuals (*F_7,98364_* = 22909, p-value < 0.001) and, due to the extremely high numbers of samples for each individual, post-hoc Tukey’s test reveals that all individuals are significantly different except for ST61 and ST65b (Fig S4D). Horse 2017-104 shows the highest ^87^Sr/^86^Sr ratio (0.7135 ± 0.0005, Fig S5). A group of horses, comprising ST61, ST63, ST65a, ST65b and ST66, follows at 0.7117 (± 0.0019) with a difference between the averaged ^87^Sr/^86^Sr ratio of these individuals below 0.0005 (Fig S5 and S4D). Finally, ST67 (0.7108 ± 0.0003) then ST62 (0.7085 ± 0.0024) present the lowest ^87^Sr/^86^Sr ratios (Figure S5).

**
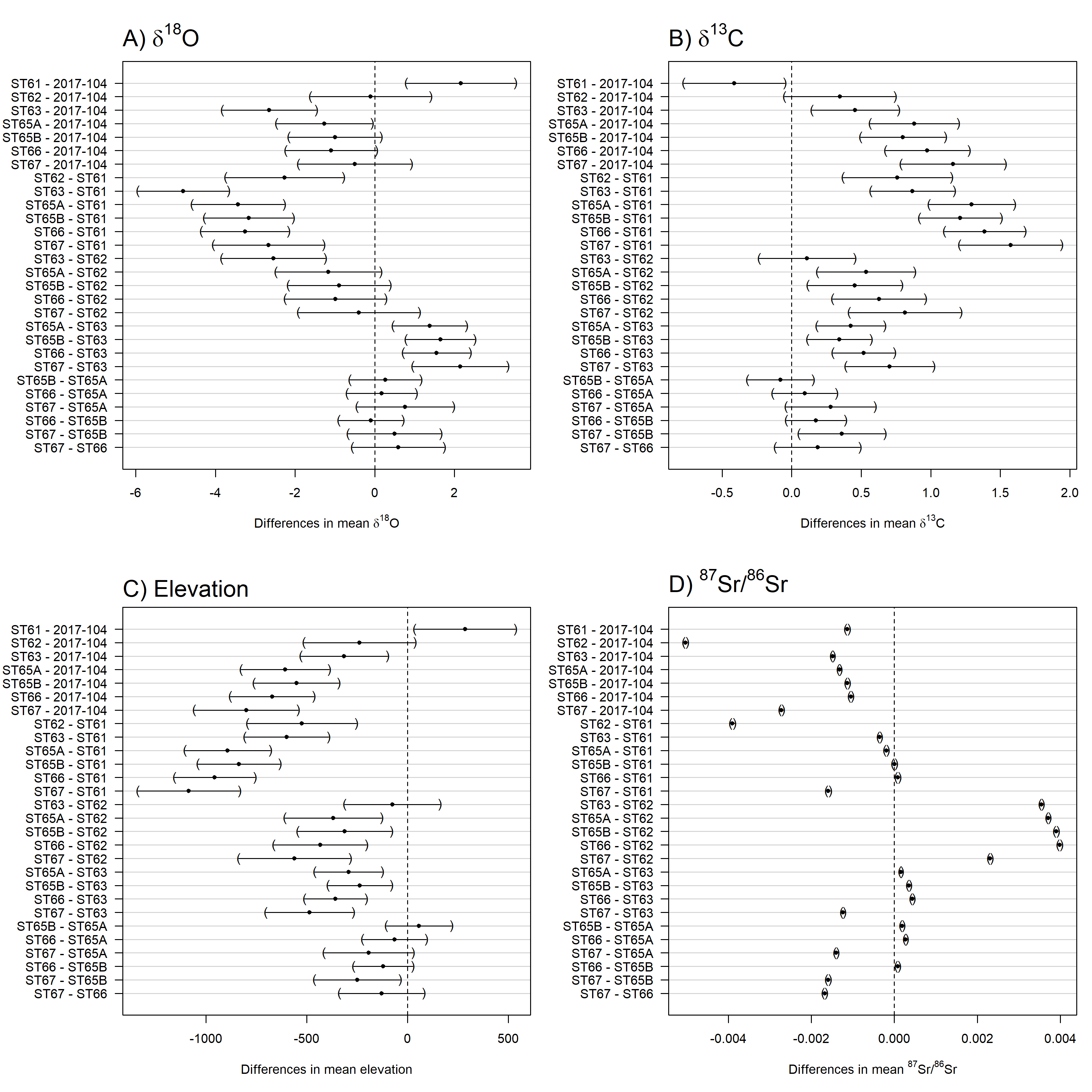
Figure S2. Tukey’s post-hoc pairewise comparisons between the seven Bronze age horses from Burgast and the modern horse 2017-104.** A) δ^18^O, B) δ^13^C, C) elevation and D) ^87^Sr/^86^Sr. Difference in mean between two individuals is presented with the 95% confidence intervals (95% CI). The difference is considered as not significant when the 95% CI overlap 0.

**
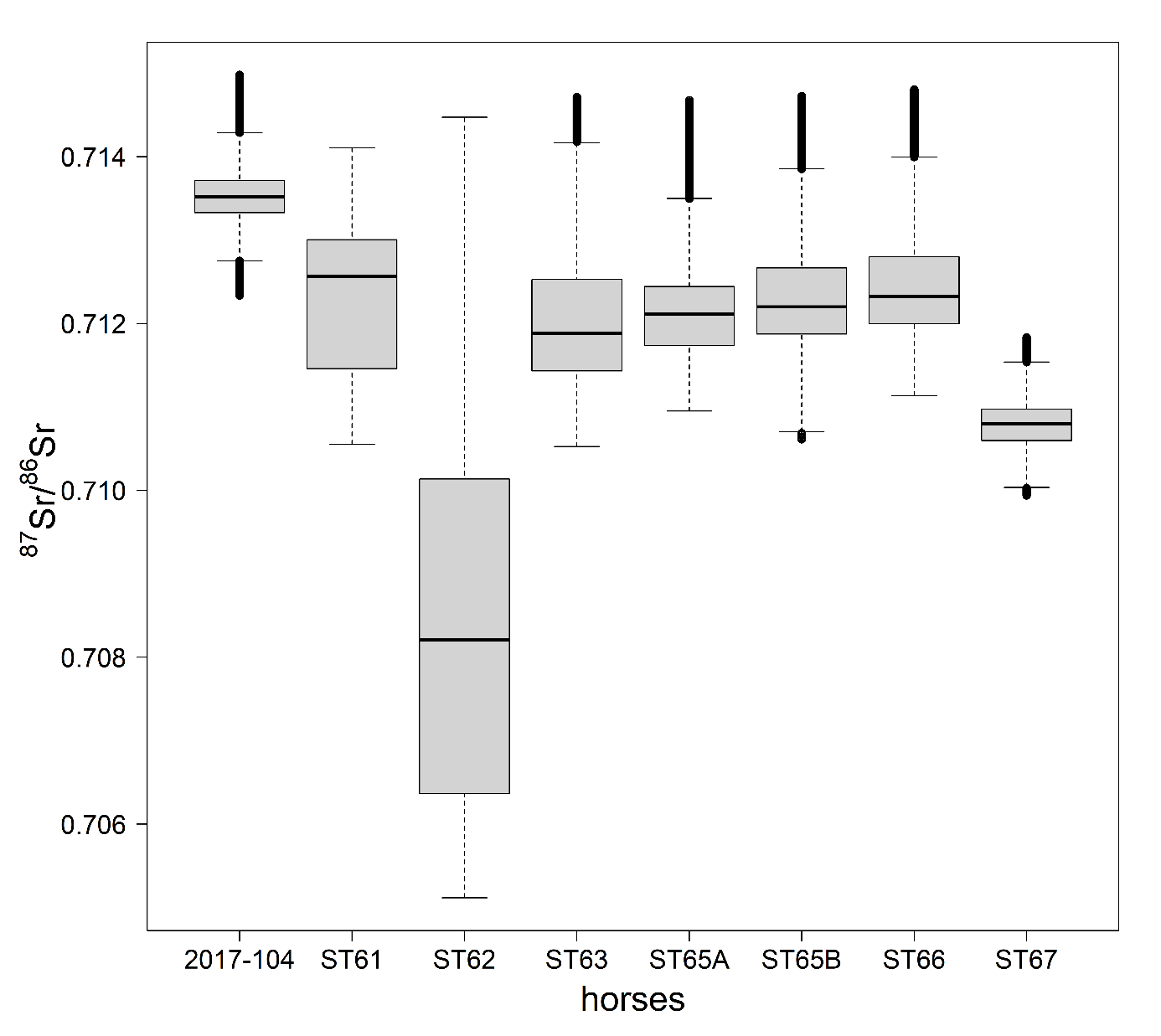
Figure S3. Differences in ^87^Sr/^86^Sr values between the seven Bronze age horses from Burgast and the modern horse 2017-104.**

**Table S5. Descriptive statistics of the intra-tooth δ^18^O (‰ V-PDB) and δ^13^C (‰ V-PDB) profiles of the seven Bronze Age horses from the Burgast site, Altai, Mongolia, and the modern horse (2017-104) from the same region.** Significant correlations between δ^18^O and δ^13^C are indicated in bold.

|  |  | δ^18^O | | | | | δ^13^C | | | | | Pearson’s correlation | |
| --- | --- | --- | --- | --- | --- | --- | --- | --- | --- | --- | --- | --- | --- |
| Individual | teeth | mean | std.dev | min | max | range | mean | std.dev | min | max | range | *r* | p.value |
| ST61 | M_1_^(a)^ | -11.63 | 2.02 | -13.7 | -6.8 | 6.9 | -12.89 | 0.40 | -14.0 | -12.4 | 1.6 | 0.15 | 0.528 |
| ST61 | M_2_^(a)^ | -6.94 | 1.00 | -9.2 | -5.7 | 3.5 | -14.94 | 0.30 | -15.4 | -14.6 | 0.8 | -0.27 | 0.344 |
| ST62 | M_1_ | -12.26 | 1.25 | -13.9 | -10.0 | 3.9 | -13.10 | 0.24 | -13.5 | -12.7 | 0.8 | -0.55 | 0.159 |
| ST62 | M_2_ | -12.40 | 0.87 | -13.5 | -10.5 | 3.0 | -13.17 | 0.64 | -13.9 | -11.8 | 2.1 | 0.07 | 0.850 |
| ST62 | M_3_ | -10.70 | 1.40 | -12.9 | -9.3 | 3.6 | -12.63 | 0.27 | -12.9 | -12.2 | 0.7 | -0.64 | 0.175 |
| ST63 | M_1_ | -14.45 | 2.02 | -17.7 | -11.0 | 6.7 | -13.22 | 0.38 | -13.8 | -12.3 | 1.5 | -0.33 | 0.134 |
| ST63 | M_2_ | -14.84 | 1.88 | -18.0 | -11.5 | 6.5 | -12.79 | 0.39 | -13.5 | -11.7 | 1.8 | 0.24 | 0.239 |
| ST63 | M_3_ | -14.06 | 1.76 | -16.6 | -10.3 | 6.3 | -12.73 | 0.52 | -13.3 | -10.7 | 2.6 | -0.11 | 0.602 |
| ST65A | M^1^ | -12.79 | 0.91 | -14.6 | -10.8 | 3.8 | -12.47 | 0.36 | -13.2 | -11.8 | 1.4 | -0.22 | 0.331 |
| ST65A | M^2^ | -13.25 | 1.62 | -15.5 | -10.2 | 5.3 | -12.44 | 0.34 | -13.0 | -11.9 | 1.1 | **-0.73** | **0.000** |
| ST65A | M^3^ | -13.18 | 1.50 | -15.5 | -11.1 | 4.4 | -12.50 | 0.33 | -13.0 | -11.6 | 1.4 | -0.18 | 0.415 |
| ST65B | M_1_ | -12.32 | 1.70 | -15.4 | -9.9 | 5.5 | -12.51 | 0.13 | -12.7 | -12.3 | 0.4 | **-0.42** | **0.031** |
| ST65B | M_2_ | -13.46 | 1.88 | -16.1 | -10.6 | 5.5 | -12.67 | 0.21 | -13.0 | -12.2 | 0.8 | -0.34 | 0.055 |
| ST65B | M_3_ | -12.51 | 1.72 | -16.0 | -10.6 | 5.4 | -12.46 | 0.38 | -13.6 | -11.8 | 1.8 | -0.29 | 0.137 |
| ST66 | M^1^ | -12.79 | 1.72 | -15.4 | -10.2 | 5.2 | -12.53 | 0.33 | -12.9 | -11.5 | 1.4 | **-0.42** | **0.022** |
| ST66 | M^2^ | -13.02 | 1.85 | -15.8 | -10.1 | 5.7 | -12.39 | 0.38 | -12.9 | -11.4 | 1.5 | -0.09 | 0.626 |
| ST66 | M^3(a)^ | -12.88 | 1.54 | -16.1 | -10.2 | 5.9 | -12.23 | 0.29 | -12.7 | -11.5 | 1.2 | -0.17 | 0.322 |
| ST67 | M_1_ | -12.70 | 1.32 | -14.7 | -11.1 | 3.6 | -12.39 | 0.41 | -13.3 | -11.9 | 1.4 | -0.43 | 0.243 |
| ST67 | M_2_ | -12.87 | 0.67 | -14.1 | -11.9 | 2.2 | -12.28 | 0.55 | -12.8 | -11.2 | 1.6 | 0.54 | 0.088 |
| ST67 | M_3_ | -11.22 | 0.93 | -12.4 | -9.6 | 2.8 | -11.88 | 0.53 | -12.4 | -10.9 | 1.5 | -0.33 | 0.384 |
| 2017-104 | M_2_^(a)^ | -11.80 | 1.93 | -14.7 | -7.2 | 7.5 | -13.35 | 0.35 | -14.0 | -12.8 | 1.2 | -0.26 | 0.173 |

^(a)^ δ^18^O data published in Lazzerini et al. [19]

**Table S6.** **Descriptive statistics of the elevation reflected by the δ^13^C variation within the teeth of the seven Bronze Age horses from the Burgast site, Altai, Mongolia, and the modern horse (2017-104) from the same region.** Elevation was estimated from the δ^13^C enamel values converted into δ^13^C from diet (δ^13^C_diet_).

|  |  | δ^13^C_diet_ | | Elevation (m) | | | | |
| --- | --- | --- | --- | --- | --- | --- | --- | --- |
| Individual | teeth | mean | std.dev | mean | std.dev | min | max | Range |
| ST61 | M_1_ | -26.59 | 0.40 | 1693 | 277 | 1352 | 2455 | 1103 |
| ST61 | M_2_ | -28.64 | 0.30 | 3106 | 204 | 2869 | 3421 | 552 |
| ST62 | M_1_ | -26.80 | 0.24 | 1834 | 169 | 1559 | 2110 | 551 |
| ST62 | M_2_ | -26.87 | 0.64 | 1880 | 443 | 938 | 2386 | 1448 |
| ST62 | M_3_ | -26.33 | 0.27 | 1513 | 183 | 1214 | 1697 | 483 |
| ST63 | M_1_ | -26.92 | 0.38 | 1916 | 259 | 1283 | 2317 | 1034 |
| ST63 | M_2_ | -26.49 | 0.39 | 1620 | 270 | 869 | 2110 | 1241 |
| ST63 | M_3_ | -26.43 | 0.52 | 1577 | 358 | 179 | 1972 | 1793 |
| ST65A | M^1^ | -26.17 | 0.36 | 1399 | 248 | 938 | 1903 | 965 |
| ST65A | M^2^ | -26.14 | 0.34 | 1380 | 234 | 1007 | 1766 | 759 |
| ST65A | M^3^ | -26.20 | 0.33 | 1421 | 230 | 800 | 1766 | 966 |
| ST65B | M_1_ | -26.21 | 0.13 | 1426 | 87 | 1283 | 1559 | 276 |
| ST65B | M_2_ | -26.37 | 0.21 | 1535 | 145 | 1214 | 1766 | 552 |
| ST65B | M_3_ | -26.16 | 0.38 | 1394 | 260 | 938 | 2179 | 1241 |
| ST66 | M^1^ | -26.23 | 0.33 | 1445 | 226 | 731 | 1697 | 966 |
| ST66 | M^2^ | -26.09 | 0.38 | 1348 | 265 | 662 | 1697 | 1035 |
| ST66 | M^3^ | -25.93 | 0.29 | 1237 | 201 | 731 | 1559 | 828 |
| ST67 | M_1_ | -26.09 | 0.41 | 1344 | 283 | 1007 | 1972 | 965 |
| ST67 | M_2_ | -25.98 | 0.55 | 1270 | 379 | 524 | 1628 | 1104 |
| ST67 | M_3_ | -25.58 | 0.53 | 992 | 363 | 317 | 1352 | 1035 |
| 2017-104 | M_2_ | -27.05 | 0.35 | 2007 | 243 | 1629 | 2453 | 824 |

**Table S7.** **Descriptive statistics of the intra-tooth ^87^Sr/^86^Sr profiles of the seven Bronze Age horses from the Burgast site, Altai, Mongolia, and the modern horse (2017-104) from the same region.**

| Individual | teeth | mean | std.dev | minimum | maximum | range |
| --- | --- | --- | --- | --- | --- | --- |
| ST61 | M_1_ | 0.7124 | 0.0009 | 0.7106 | 0.7140 | 0.0034 |
| ST62 | M_1_ | 0.7065 | 0.0008 | 0.7052 | 0.7084 | 0.0032 |
| ST62 | M_2_ | 0.7081 | 0.0019 | 0.7054 | 0.7118 | 0.0064 |
| ST62 | M_3_ | 0.7114 | 0.0015 | 0.7094 | 0.7143 | 0.0049 |
| ST63 | M_2_ | 0.7120 | 0.0009 | 0.7107 | 0.7146 | 0.0040 |
| ST63 | M_3_ | 0.7121 | 0.0007 | 0.7107 | 0.7137 | 0.0031 |
| ST65A | M^2^ | 0.7123 | 0.0005 | 0.7115 | 0.7135 | 0.0020 |
| ST65A | M^3^ | 0.7121 | 0.0008 | 0.7110 | 0.7146 | 0.0035 |
| ST65B | M_2_ | 0.7124 | 0.0008 | 0.7109 | 0.7146 | 0.0037 |
| ST66 | M^2^ | 0.7125 | 0.0008 | 0.7112 | 0.7147 | 0.0035 |
| ST67 | M_1_ | 0.7109 | 0.0004 | 0.7103 | 0.7118 | 0.0016 |
| ST67 | M_2_ | 0.7107 | 0.0003 | 0.7100 | 0.7114 | 0.0014 |
| ST67 | M_3_ | 0.7108 | 0.0002 | 0.7103 | 0.7116 | 0.0013 |
| 2017-104 | M_2_ | 0.7135 | 0.0005 | 0.7123 | 0.7150 | 0.0027 |

**Supplementary material SM6. Spatial assignments.**


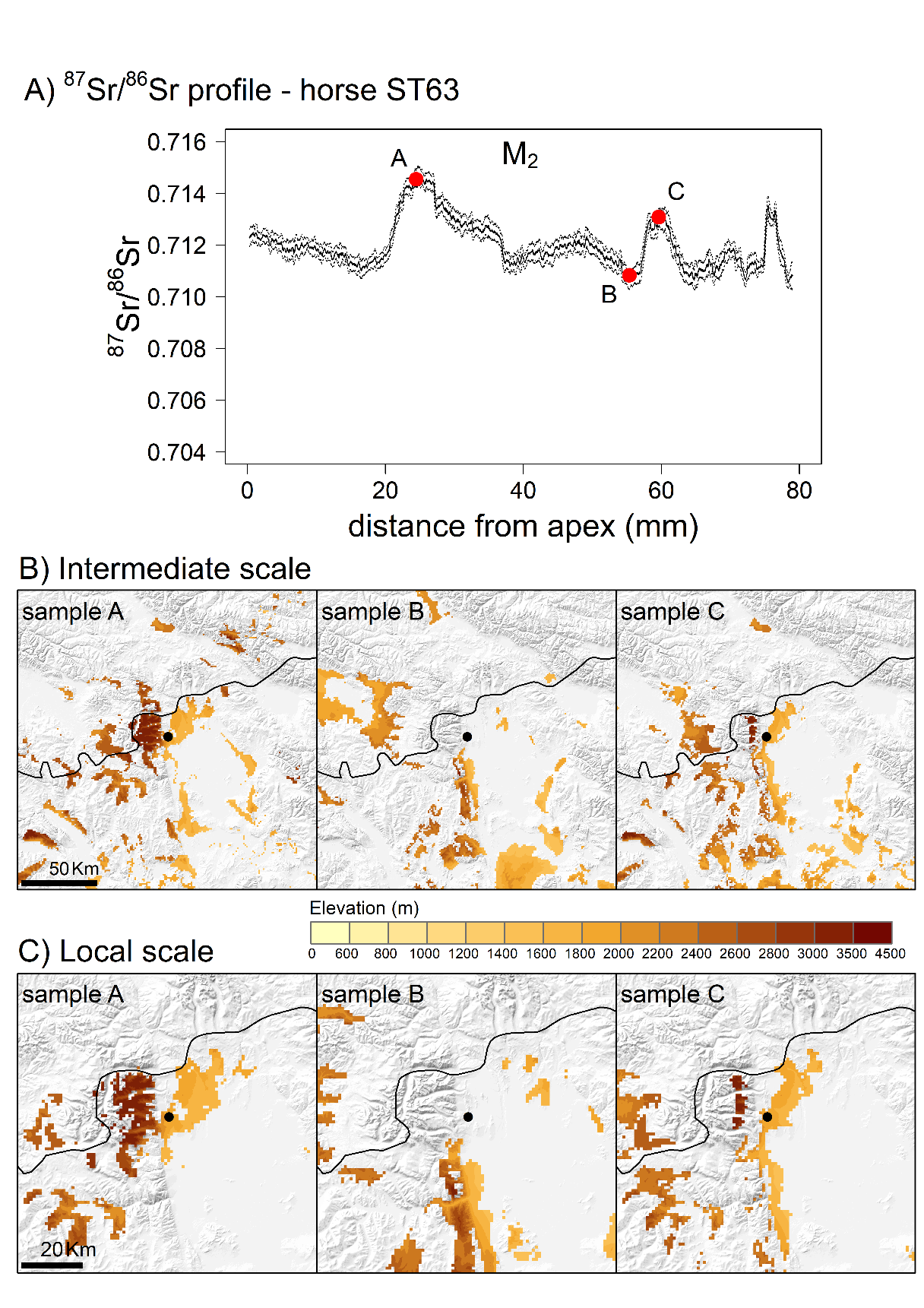


**Figure S4. Geographic assignment of ^87^Sr/^86^Sr enamel samples from the Late Bronze Age horse ST63**. A) ^87^Sr/^86^Sr intra-tooth profile and the 3 samples selected for assignment (red dot), B) the assignment maps of the 3 samples on an area of 200*200 km centered on the archaeological site of Burgast (black dot), and C) the assignment maps of the 3 samples on an area of 100*100 km centered on Burgast.  Elevation is displayed within each assigned area in brown color scale. Assignments correspond to the 10% of the assignment map surface with the highest probability of origin.


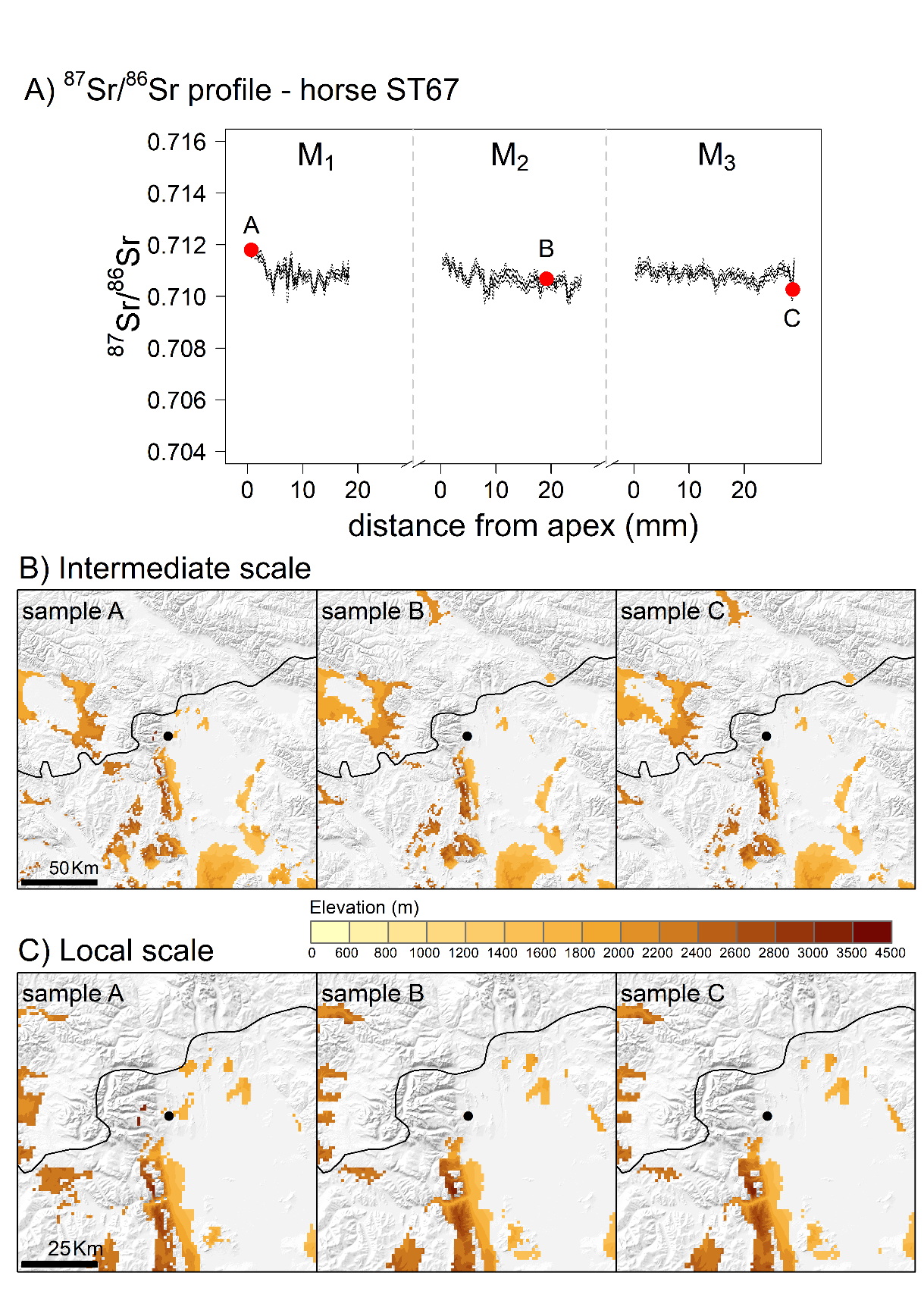


**Figure S5. Geographic assignment of ^87^Sr/^86^Sr enamel samples from the Late Bronze Age horse ST67.** A) ^87^Sr/^86^Sr intra-tooth profile and the 3 samples selected for assignment (red dot), B) the assignment maps of the 3 samples on an area of 200*200 km centered on the archaeological site of Burgast (black dot), and C) the assignment maps of the 3 samples on an area of 100*100 km centered on Burgast. Elevation is displayed within each assigned area in brown color scale. Assignments correspond to the 10% of the assignment map surface with the highest probability of origin.

**
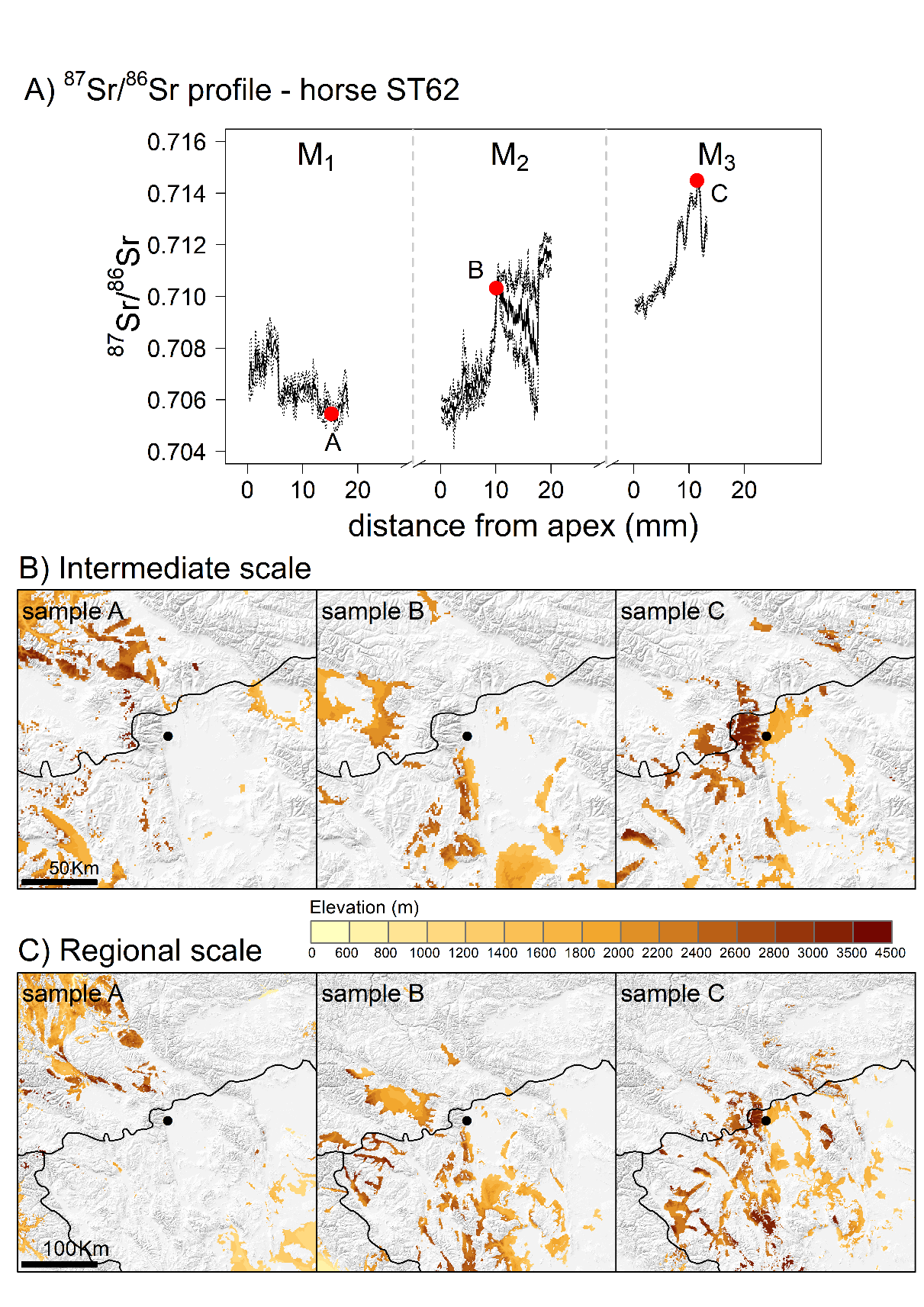
**

**Figure S6. Geographic assignment of ^87^Sr/^86^Sr enamel samples from the Late Bronze Age horse ST62.** A) ^87^Sr/^86^Sr intra-tooth profile and the 3 samples selected for assignment (red dot), B) the assignment maps of the 3 samples on an area of 200*200 km centered on the archaeological site of Burgast (black dot), and C) the assignment maps of the 3 samples on an area of 400*400 km centered on Burgast. Elevation is displayed within each assigned area in brown color scale. Assignments correspond to the 10% of the assignment map surface with the highest probability of origin.

**
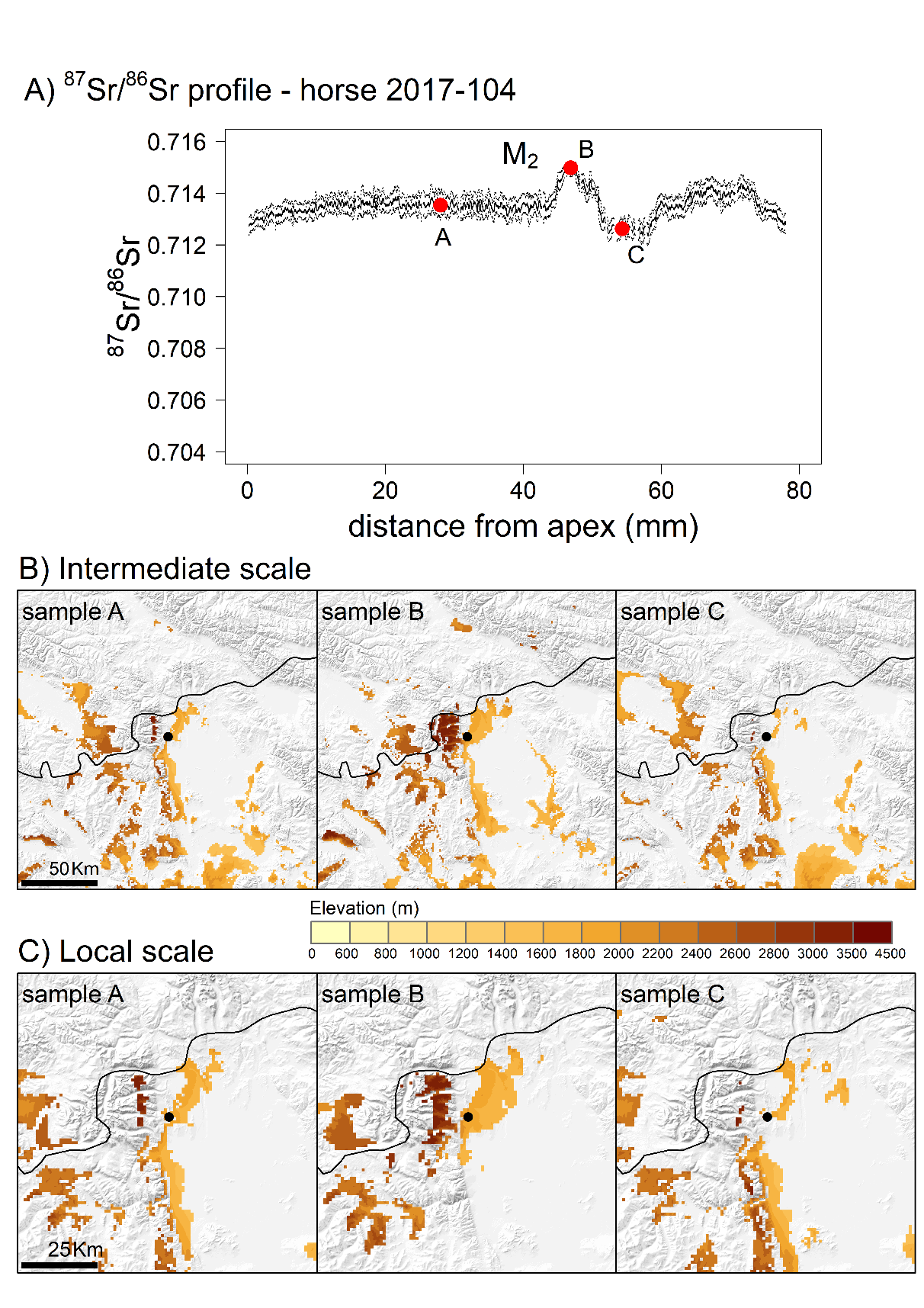
**

**Figure S7. Geographic assignment of ^87^Sr/^86^Sr enamel samples from modern horse 2017-104.** A) ^87^Sr/^86^Sr intra-tooth profile and the 3 samples selected for assignment (red dot), B) the assignment maps of the 3 samples on an area of 200*200 km centered on the archaeological site of Burgast (black dot), and C) the assignment maps of the 3 samples on an area of 100*100 km centered on Burgast. Elevation is displayed within each assigned area in brown color scale. Assignments correspond to the 10% of the assignment map surface with the highest probability of origin.


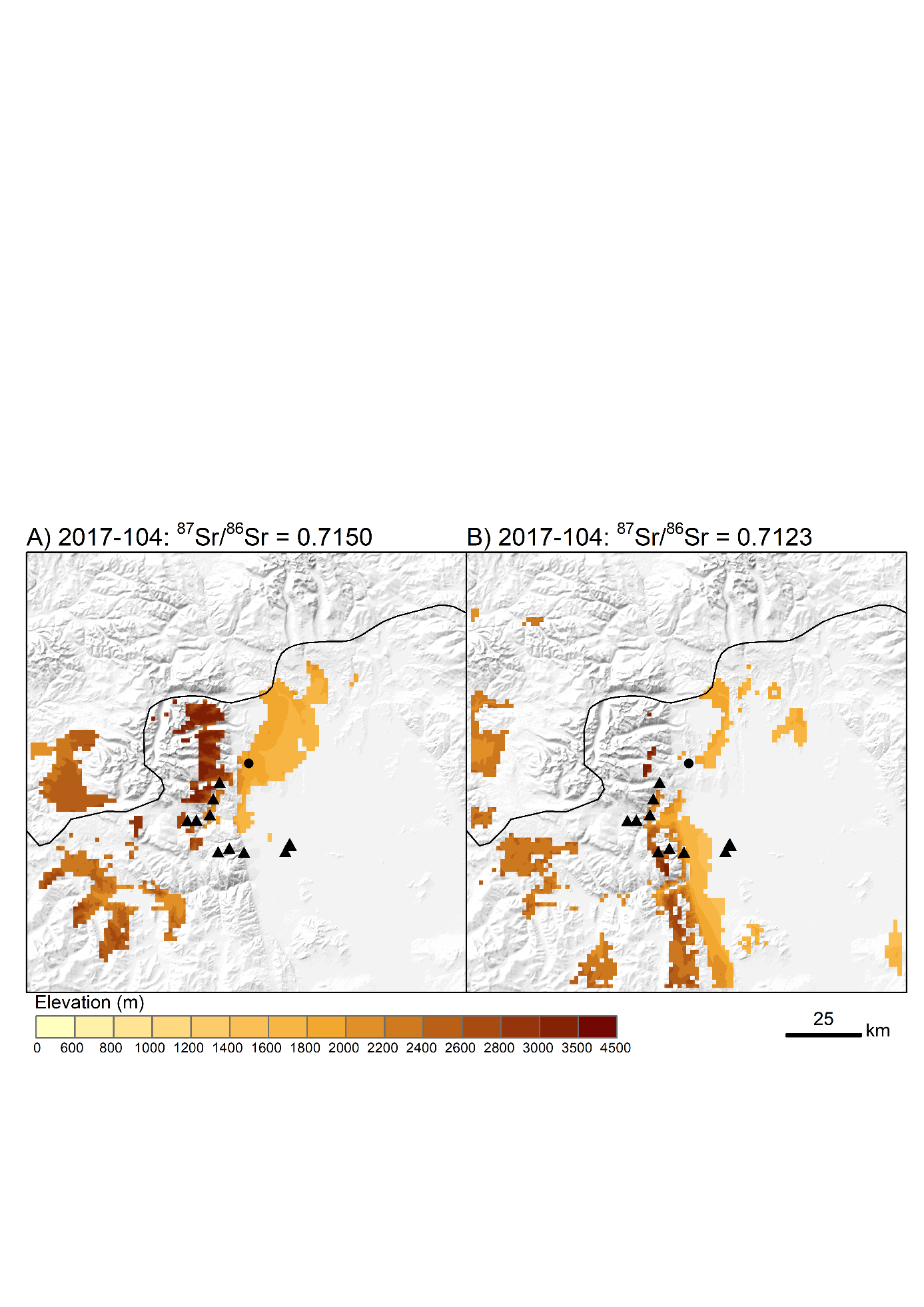


**Figure S8. Geographic assignment of the modern horse 2017-104.** Assignment for A) the highest value and B) the lowest value of the ^87^Sr/^86^Sr tooth enamel profile. The herder family's camp locations are indicated by the black triangles and the Burgast archaeological site by the black dot. Elevation is displayed within each assigned area in brown color scale.


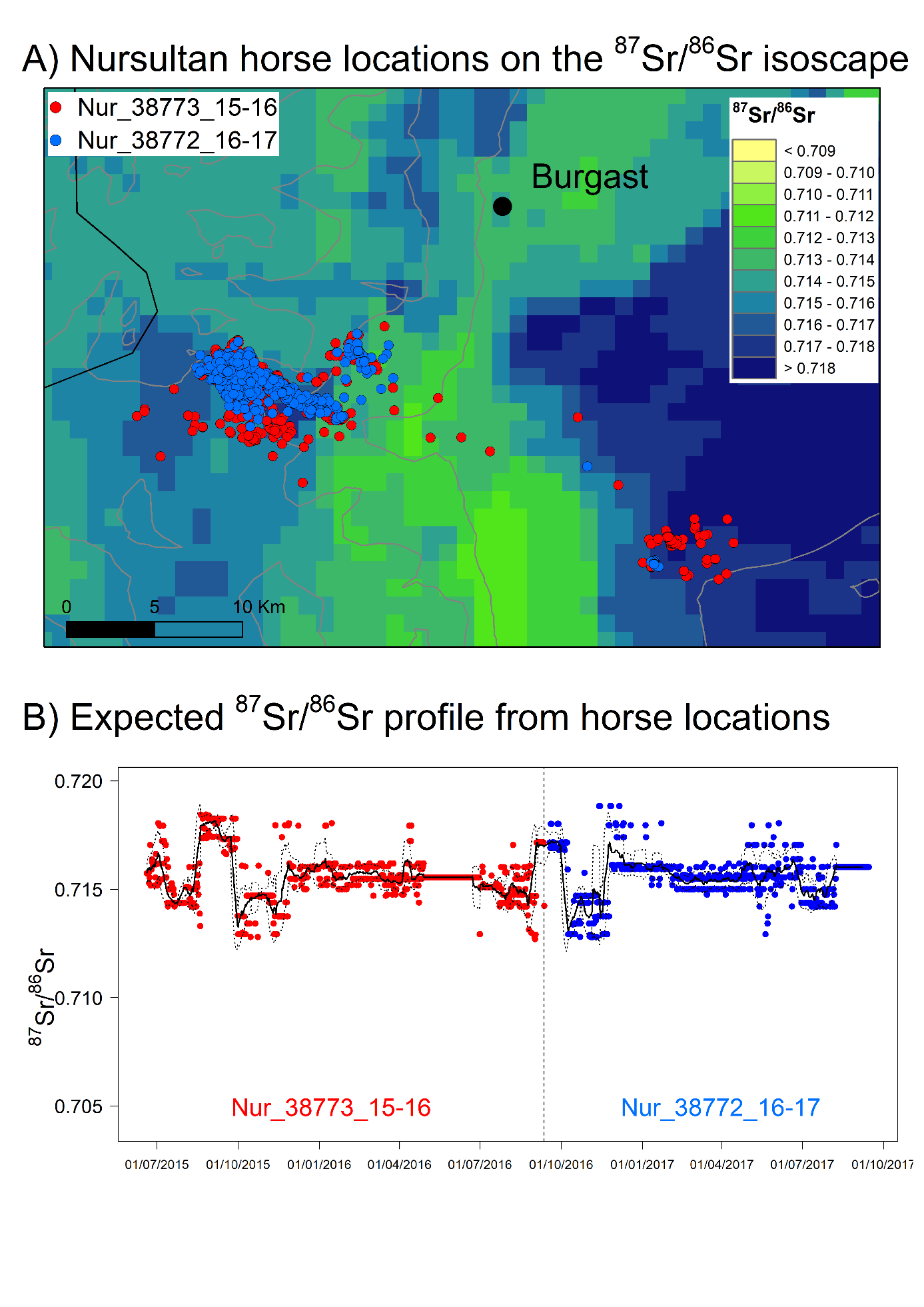


**Figure S9.** **Expected ^87^Sr/^86^Sr profile from horse movements registered by GPS collar.** A) GPS locations of Nursultan's family's horse on the ^87^Sr/^86^Sr isoscape over two years. B) Expected ^87^Sr/^86^Sr profile with ^87^Sr/^86^Sr from the isoscape sampled at each GPS location.

**References**

1. Bataille CP, Crowley BE, Wooller MJ, Bowen GJ. Advances in global bioavailable strontium isoscapes. Palaeogeogr Palaeoclimatol Palaeoecol. 2020;555: 109849. doi:10.1016/j.palaeo.2020.109849

2. Le Corre M, Dargent F, Grimes V, Wrigth J, Côté SD, Reich MS, et al. An ensemble machine learning bioavailable strontium isoscape for Eastern Canada. FACETS. 2024.

3. Lazzerini N, Balter V, Coulon A, Tacail T, Marchina C, Lemoine M, et al. Monthly mobility inferred from isoscapes and laser ablation strontium isotope ratios in caprine tooth enamel. Sci Rep. 2021;11. doi:10.1038/s41598-021-81923-z

4. Bataille CP, Brennan SR, Hartmann J, Moosdorf N, Wooller MJ, Bowen GJ. A geostatistical framework for predicting variations in strontium concentrations and isotope ratios in Alaskan rivers. Chem Geol. 2014;389: 1–15. doi:10.1016/j.chemgeo.2014.08.030

5. R core Team. R: A language and environment for statistical computing. R Foundation for Statistical Computing; 2023.

6. Breiman L. Random Forests. Mach Learn. 2001;45: 5–32. doi:10.1023/A:1010933404324

7. Genuer R, Poggi J-M, Tuleau-Malot C. VSURF: An R Package for Variable Selection Using Random Forests. R J. 2015;7: 19–33.

8. Funck J, Bataille C, Rasic J, Wooller M. A bio‐available strontium isoscape for eastern Beringia: a tool for tracking landscape use of Pleistocene megafauna. J Quat Sci. 2021;36: 76–90. doi:10.1002/jqs.3262

9. Hartmann J, Moosdorf N. The new global lithological map database GLiM: A representation of rock properties at the Earth surface. Geochemistry, Geophysics, Geosystems. 2012;13. doi:10.1029/2012GC004370

10. Mooney WD, Laske G, Masters TG. CRUST 5.1: A global crustal model at 5° × 5°. J Geophys Res Solid Earth. 1998;103: 727–747. doi:10.1029/97JB02122

11. Hijmans RJ, Cameron SE, Parra JL, Jones PG, Jarvis A. Very high resolution interpolated climate surfaces for global land areas. International Journal of Climatology. 2005;25: 1965–1978. doi:10.1002/joc.1276

12. Zomer RJ, Trabucco A, Bossio DA, Verchot L V. Climate change mitigation: A spatial analysis of global land suitability for clean development mechanism afforestation and reforestation. Agric Ecosyst Environ. 2008;126: 67–80. doi:10.1016/j.agee.2008.01.014

13. Vet R, Artz RS, Carou S, Shaw M, Ro C-U, Aas W, et al. A global assessment of precipitation chemistry and deposition of sulfur, nitrogen, sea salt, base cations, organic acids, acidity and pH, and phosphorus. Atmos Environ. 2014;93: 3–100. doi:10.1016/j.atmosenv.2013.10.060

14. Mahowald NM, Muhs DR, Levis S, Rasch PJ, Yoshioka M, Zender CS, et al. Change in atmospheric mineral aerosols in response to climate: Last glacial period, preindustrial, modern, and doubled carbon dioxide climates. Journal of Geophysical Research: Atmospheres. 2006;111: D1020. doi:10.1029/2005JD006653

15. Jarvis A, Reuter A, Nelson A, Guevara E. Hole-filled SRTM for the globe Version 4, available from the CGIAR-CSI SRTM 90m Database. In: CGIAR CSI Consort Spat Inf [Internet]. 2008 [cited 9 Oct 2023] pp. 1–9. Available: http://srtm.csi.cgiar.org

16. Balmino G, Vales N, Bonvalot S, Briais A. Spherical harmonic modelling to ultra-high degree of Bouguer and isostatic anomalies. J Geod. 2012;86: 499–520. doi:10.1007/s00190-011-0533-4

17. Börker J, Hartmann J, Amann T, Romero‐Mujalli G. Terrestrial sediments of the Earth: Development of a Global Unconsolidated sediments Map database (GUM). Geochemistry, Geophysics, Geosystems. 2018;19: 997–1024. doi:10.1002/2017GC007273

18. Poggio L, de Sousa LM, Batjes NH, Heuvelink GBM, Kempen B, Ribeiro E, et al. SoilGrids 2.0: producing soil information for the globe with quantified spatial uncertainty. SOIL. 2021;7: 217–240. doi:10.5194/soil-7-217-2021

19. Lazzerini N, Coulon A, Simon L, Marchina C, Fiorillo D, Turbat T, et al. The isotope record (δ^13^C, δ^18^O) of vertical mobility in incremental tissues (tooth enamel, hair) of modern livestock: A reference set from the Mongolian Altai. Quaternary International. 2021;595: 128–144. doi:10.1016/j.quaint.2021.04.008
