# Supplementary material for: 3000 yr-old patterns of mobile pastoralism revealed by multiple isotopes and radiocarbon dating of ancient horses from the Mongolian Altai
Source: PLoS One. 2025 May 7;20(5):e0322431. doi: 10.1371/journal.pone.0322431 (PMC12057990; doi:10.1371/journal.pone.0322431)
Supplement: S5 File — Isoscape and geographic assignment R scripts. (DOCX) [file pone.0322431.s005.docx]

################################################################################

######## GENERATING SR ISOSCAPE FOR MONGOLIA USING RANDOM FOREST ##############

################################################################################

# The script is adapated from the original script of Bataille CP, Crowley BE, Wooller MJ and GJ Bowen. 2020.

# Advances in global bioavailable strontium isoscapes. Palaeogeography, Palaeoclimatology, Palaeoecology, 555: 109849.

# DOI: https://doi.org/10.1016/j.palaeo.2020.109849

###for geographic assignment, see line 350

setwd("D:/.../RF_isoscape")

##########################SET LIBRARIES##################################################

library(parallel)

library(doParallel)

library(raster)

library(terra)

library(sf)

library(randomForest)

library(readxl)

library(exactextractr)

library(caret)

library(VSURF)

library(ranger)

# Choose what is relevant for your OS

os="unix" # this includes osx on a Mac

os="win"

# Helper function to store (print) files in sub folders in different OS's

os_path <- function(folder_path,filename){

if (os=="win") return(paste(folder_path,"/",filename,sep="")) else

return(paste(folder_path,"/",filename,sep="")) }

#!! Create or use sub folders to keep things tidy!

Out_Path <- "Output"

Ras_Path <- "Projected_rasters"

mydir = getwd()

dir.create(file.path(mydir,Ras_Path, fsep = .Platform$file.sep), showWarnings = FALSE)

dir.create(file.path(mydir,Out_Path, fsep = .Platform$file.sep), showWarnings = FALSE)

################################################################################

############################## DATA PROCESSING #################################

################################################################################

# load bioavailable Sr database

# this database is an updated version of the global bioavailable Sr database from Bataille et al. 2020

sr_orig <- readxl::read_excel("SM7_Table_S8_bioavailable_sr.xlsx",col_names=TRUE, na="NA", sheet="samples")

sr_orig<-as.data.frame(sr_orig)

# Clean missing values

sr_orig_0<-sr_orig[!is.na(sr_orig$Latitude),] #remove rows with missing XY coordinates

sr_orig_0<-sr_orig_0[!is.na(sr_orig$Longitude),]

sr_orig_1<-sr_orig_0[!is.na(sr_orig_0$`87Sr/86Sr`),] #remove rows with missing observed Sr data

# Project Sr data

sr_proj<-st_as_sf(sr_orig_1, coords=c("Longitude","Latitude"), crs=st_crs(4326))%>%

st_transform(crs="+proj=eck4 +lon_0=0 +x_0=0 +y_0=0 +ellps=WGS84 +units=m +no_defs")

#################################INPUT GLOBAL COVARIATE RASTERS#####################################################

###See Table 1 for raster list and references

###The projected covariates are available at:

###https://drive.google.com/drive/folders/1g9rCGo3Kd3hz2o5JKkSbgNsGJclvsuQm?usp=sharing

###or on request to Mael Le Corre (mael.lecorre@abdn.ac.uk) or Clément Bataille (cbataill@uottawa.ca)

###agemin, agemean, agemax, srsrmed, srsrq1, srsrq3 rasters are available through the data repository link provided in the paper

###Use this link and download the Projected_rasters folder as a .zip file into your working directory (35GO)

###Unzip the file using the 7zip program https://www.7-zip.org/ which can handle large .zip file.

###Verify that the unzip folder has a name of "Projected_rasters" as below and if necessary rename the folder to the correct name

###Once this is done the following raster should load in your script

r.m1 =terra::rast(os_path(Ras_Path,"srsrmed_qcupdate.tif"))

r.srsrq1 =terra::rast(os_path(Ras_Path,"srsrq1_qcupdate.tif"))

r.srsrq3 =terra::rast(os_path(Ras_Path,"srsrq3_qcupdate.tif"))

r.meanage_geol =terra::rast(os_path(Ras_Path,"agemean_qcupdate.tif"))

r.minage_geol =terra::rast(os_path(Ras_Path,"agemin_qcupdate.tif"))

r.maxage_geol =terra::rast(os_path(Ras_Path,"agemax_qcupdate.tif"))

r.age =terra::rast(os_path(Ras_Path,"basement_age_reproj.tif"))

r.bouger =terra::rast(os_path(Ras_Path,"bouger_reproj.tif"))

r.elevation =terra::rast(os_path(Ras_Path,"elevation_reproj.tif"))

r.mat =terra::rast(os_path(Ras_Path,"mat_reproj.tif"))

r.map =terra::rast(os_path(Ras_Path,"map_reproj.tif"))

r.dust =terra::rast(os_path(Ras_Path,"dust_dep_reproj.tif"))

r.salt =terra::rast(os_path(Ras_Path,"seasalt_dep_reproj.tif"))

r.ai =terra::rast(os_path(Ras_Path,"ai_reproj.tif"))

r.pet =terra::rast(os_path(Ras_Path,"pet_reproj.tif"))

r.clay =terra::rast(os_path(Ras_Path,"r.clay_reproj.tif"))

r.ph =terra::rast(os_path(Ras_Path,"r.ph_reproj.tif"))

r.cec =terra::rast(os_path(Ras_Path,"r.cec_reproj.tif"))

r.bulk =terra::rast(os_path(Ras_Path,"r.bulk_reproj.tif"))

r.GUM =terra::rast(os_path(Ras_Path,"gum_mask3.tif"))

r.phkcl =terra::rast(os_path(Ras_Path,"phkcl.tif"))

r.ocs =terra::rast(os_path(Ras_Path,"r.ocs_reproj.tif"))

# Function to extract covariables at the sampling sites or at the closest of the sampling site

# if the site fall within an empty cell

extract_nearest_non_na <- function(coordinates, raster) {

# Convert the coordinates data.frame to an sf points object

points <- st_as_sf(coordinates, coords = c("X", "Y"), crs = st_crs(raster))

# Extract raster values under the points

values <- extract(raster, points,method="simple")

values<-values[,2]

# Identify NA values

na_indices <- is.na(values)

# Find the indices of NA values

na_indices <- which(na_indices)

# Loop over each NA value

for (i in na_indices) {

# Get the current point

point <- points[i, ]

st_crs(point) <- st_crs(raster)

# Create a buffer around the point with a 50km radius

buffer <- st_buffer(point, dist = 50000)

# Convert the buffer to the same CRS as the raster

st_crs(buffer) <- st_crs(raster)

# Crop the raster to the buffered extent

cropped_raster <- crop(raster, buffer)

# Extract the non-NA values within the buffer

non_na_values <- cropped_raster[]

non_na_values <- non_na_values[!is.na(non_na_values)]

# Find the nearest non-NA value

nearest_value <- non_na_values[which.min(st_distance(point, buffer))]

# Assign the nearest non-NA value to replace the NA value

values[i] <- nearest_value

}

# Return the extracted values

return(data.frame(values))

}

# data extraction

m1xy<-extract_nearest_non_na(sr_proj,r.m1)

srsrq1xy<-extract_nearest_non_na(sr_proj,r.srsrq1)

srsrq3xy<-extract_nearest_non_na(sr_proj,r.srsrq3)

meanage_geolxy<-extract_nearest_non_na(sr_proj,r.meanage_geol)

minage_geolxy<-extract_nearest_non_na(sr_proj,r.minage_geol)

maxage_geolxy<-extract_nearest_non_na(sr_proj,r.maxage_geol)

agexy<-extract_nearest_non_na(sr_proj,r.age)

bougerxy=extract_nearest_non_na(sr_proj,r.bouger)

elevationxy<-extract_nearest_non_na(sr_proj,r.elevation)

mapxy<-extract_nearest_non_na(sr_proj,r.map)

matxy=extract_nearest_non_na(sr_proj,r.mat)

dustxy<-extract_nearest_non_na(sr_proj,r.dust)

saltxy<-extract_nearest_non_na(sr_proj,r.salt)

aixy<-extract_nearest_non_na(sr_proj,r.ai)

petxy<-extract_nearest_non_na(sr_proj,r.pet)

clayxy<-extract_nearest_non_na(sr_proj,r.clay)

phxy<-extract_nearest_non_na(sr_proj,r.ph)

cecxy<-extract_nearest_non_na(sr_proj,r.cec)

bulkxy<-extract_nearest_non_na(sr_proj,r.bulk)

GUMxy<-extract_nearest_non_na(sr_proj,r.GUM)

phkclxy<-extract_nearest_non_na(sr_proj,r.phkcl)

ocsxy<-extract_nearest_non_na(sr_proj,r.ocs)

sr_xy<- data.frame(st_coordinates(sr_proj))

### Append all extracted data and change names of column

sr_proj_xy <- data.frame(sr_orig_1$ID,sr_orig_1$Country,sr_orig_1$Latitude,sr_orig_1$Longitude, sr_orig_1$`87Sr/86Sr`,

sr_xy,m1xy,srsrq1xy,srsrq3xy,meanage_geolxy,minage_geolxy,maxage_geolxy,

agexy,bougerxy,elevationxy,

matxy,mapxy,dustxy,saltxy,aixy,petxy,

clayxy,phxy,cecxy,bulkxy,GUMxy,phkclxy,ocsxy)

colnames(sr_proj_xy)<-c("ID","Country","Latitude","Longitude","X87Sr86Sr",

"X","Y","r.m1","r.srsrq1","r.srsrq3","r.meanage_geol","r.minage_geol","r.maxage_geol",

"r.age","r.bouger","r.elevation",

"r.mat","r.map","r.dust","r.salt","r.ai","r.pet",

"r.clay","r.ph","r.cec","r.bulk","r.GUM","r.phkcl","r.ocs")

write.table(sr_proj_xy,"sr_proj_xy.txt",sep="\t",col.names=T,row.names=F,quote=F)

#sr_proj_xy<-read.table("sr_proj_xy_alldata.txt",sep="\t",h=T)

# compute mean Sr value for each location

sr_proj_xy2<-sr_proj_xy[!is.na(sr_proj_xy$r.m1),]

sr_agg1<-aggregate(sr_proj_xy2,by=list(sr_proj_xy2$Latitude,sr_proj_xy2$Longitude), FUN=median,na.rm=TRUE)

sr_agg1<-cbind(sr_agg1[,c(8,9,7)],sr_agg1[,10:ncol(sr_agg1)])

# check correlation between covariates

round(cor(sr_agg1[,-(1:3)]),2)

sr_agg<-sr_agg1[,-c(4,7)] #remove r.m1 and meanage_geol, because R>0.9

write.table(sr_agg,"sr_agg.txt",sep="\t",col.names=T,row.names=F,quote=F) #save data with all samples

################################################################################

############################# RANDOM FOREST ####################################

################################################################################

sr_agg<-read.table("sr_agg.txt",sep="\t",h=T)

###Variable filtering using parallelized VSURF algorithm

names(sr_agg)

set.seed(1)

sr_agg0<-sr_agg

sr_agg1.vsurf<-VSURF(sr_agg0[,4:ncol(sr_agg0)],sr_agg0$X87Sr86Sr, RFimplem = "ranger", parallel = TRUE, ncores = detectCores() - 1, clusterType = "PSOCK")

sr_agg1.vsurf$varselect.pred

sr_agg1.sub<-sr_agg0[,4:ncol(sr_agg0)]

sr_agg1_VSURF <- sr_agg1.sub[c(sr_agg1.vsurf$varselect.pred)]

sr_agg1_VSURF<-cbind(sr_agg[,1:3],sr_agg1_VSURF)

training_rf <- sr_agg1_VSURF[,-c(1,2)]

###Parallelize random forest modeling

cluster <-parallel::makeCluster(detectCores() - 1) # convention to leave 1 core for OS

registerDoParallel(cluster)

# Splitting the data for repeated cross validation

fitControl <- trainControl(## 10-fold Crossvalidation

method = "repeatedcv",

number = 10,

## repeated ten times

repeats = 5,

verboseIter=FALSE ,

returnResamp="final",

savePredictions="all",

# With parallel backend

allowParallel=TRUE

)

bestmtry <- tuneRF(training_rf, training_rf$X87Sr86Sr, stepFactor=1, improve=1e-7, ntree=1000)

mtry <- bestmtry[1]

tunegrid <- expand.grid(.mtry=mtry)

metric<-"Accuracy"

# Random forest analysis

# variables selected : "r.minage_geol","r.maxage_geol","r.srsrq1","r.mat",r.pet","r.map","r.salt","r.dust"

set.seed(1)

RF_mod <- train(X87Sr86Sr ~ ., data = training_rf, ntree=3000,method = "rf", importance=TRUE, tuneGrid=tunegrid,trControl= fitControl)

save(RF_mod,file="RF_mod")

###variable importance and partial dependance plots

ly<-matrix(c(1,1,1,0,2,2,3,3,4,4,

1,1,1,0,2,2,3,3,4,4,

1,1,1,0,5,5,6,6,7,7,

1,1,1,0,5,5,6,6,7,7,

1,1,1,0,8,8,9,9,0,0,

1,1,1,0,8,8,9,9,0,0),

6,10,byrow=T)

layout(ly)

par(mar=c(5,4,2,0))

varImpPlot(RF_noec$finalModel,type=2,main="Variable Importance")

mtext("A)",cex=1.5,side=3,at=-0.0055)

mtext("B)",cex=1.5,side=3,at=0.03)

mtext(expression(paste(""^87,"Sr/"^86,"Sr predicted")),side=4,cex=1.5,line=4)

par(mar=c(4,2,1,1))

partialPlot(RF_noec$finalModel, training_rf, x.var = "r.srsrq1",main=NA,xlab="r.srsrq1",cex.axis=0.9,ylim=c(0.710,0.716))

partialPlot(RF_noec$finalModel, training_rf, x.var = "r.minage_geol",main=NA,xlab="r.minage_geol",cex.axis=0.9,ylim=c(0.710,0.716))

partialPlot(RF_noec$finalModel, training_rf, x.var = "r.maxage_geol",main=NA,xlab="r.maxage_geol",cex.axis=0.9,ylim=c(0.710,0.716))

partialPlot(RF_noec$finalModel, training_rf, x.var = "r.pet",main=NA,xlab="r.pet",cex.axis=0.9,ylim=c(0.710,0.716))

partialPlot(RF_noec$finalModel, training_rf, x.var = "r.mat",main=NA,xlab="r.mat",cex.axis=0.9,ylim=c(0.710,0.716))

partialPlot(RF_noec$finalModel, training_rf, x.var = "r.map",main=NA,xlab="r.map",cex.axis=0.9,ylim=c(0.710,0.716))

partialPlot(RF_noec$finalModel, training_rf, x.var = "r.dust",main=NA,xlab="r.dust",cex.axis=0.9,ylim=c(0.710,0.716))

partialPlot(RF_noec$finalModel, training_rf, x.var = "r.salt",main=NA,xlab="r.salt",cex.axis=0.9,ylim=c(0.710,0.716))

savePlot("Output\\importance_pplot.tif",type="tif")

###prediction map

stack_ras<-c(r.srsrq1,r.mat,r.maxage_geol,r.minage_geol,r.pet,r.salt,r.dust,r.map)

names(stack_ras)<-c("r.srsrq1","r.mat","r.maxage_geol","r.minage_geol","r.pet","r.salt","r.dust","r.map")

ex<-ext(5400000,10300000,4450000,7000000)

stack_ras<-crop(stack_ras, ex, snap='near')

mon.grid<-stack_ras[[1]]/stack_ras[[1]]

rf1 <- predict(stack_ras, RF_mod, ext=mon.grid, na.rm=TRUE, overwrite=TRUE)

writeRaster(rf1, filename="Output\\sr_isoscape_pred.tif", overwrite=TRUE)

###spatial uncertainty using Quantile regression forest

training_qrf<-read.table("sr_agg.txt",sep="\t",h=T)

qrf_mod <- ranger(X87Sr86Sr ~ r.srsrq1+r.mat+r.maxage_geol+r.minage_geol+r.pet+r.salt+r.dust+r.map,

data = training_qrf, quantreg=TRUE, num.trees=3000,mtry=3) #mtry same as for RF

# dataset need to be divided in several part to avoid memory problems

stack_ras2<-raster::stack(stack_ras)

mon_pxl<-as(stack_ras2,"SpatialPixelsDataFrame")

newdat0<-as.data.frame(mon_pxl)

cc<-which(complete.cases(mon_pxl@data))

lcc<-length(cc)

cc_div<-c(seq(0,lcc,100000),lcc) #set size of the sub-dataset, need to be changed according available memory

tab_pred<-as.data.frame(matrix(data=NA,ncol=3,nrow=0))

for (i in 1:(length(cc_div)-1))

{

print(paste(i,(length(cc_div)-1),Sys.time(),sep=" / "))

cc_sub<-cc[(cc_div[i]+1):cc_div[i+1]]

newdat1<-newdat0[cc_sub,]

sr.rfd_low <- predict(qrf_mod, data=newdat1, type="quantiles", quantiles=0.159, fun = function(model, ...) predict(model, ...)$predictions)

gc()

sr.rfd_high <- predict(qrf_mod, data=newdat1, type="quantiles", quantiles=0.841, fun = function(model, ...) predict(model, ...)$predictions)

gc()

pred<-cbind(cc_sub,sr.rfd_low$predictions,sr.rfd_high$predictions)

tab_pred<-rbind(tab_pred,pred)

rm(sr.rfd_low)

rm(sr.rfd_high)

rm(pred)

}

colnames(tab_pred)<-c("pxlnum","pred_low","pred_high")

tab_pred$error<-(tab_pred[,3]-tab_pred[,2])/2

mon_pxl@data$se.sr<-NA

mon_pxl@data$se.sr[cc]<-tab_pred$error

rast.se<-raster(EC_pxl[,"se.sr"])

writeRaster(rast.se, filename="Output\\sr_isocape_sd.tif", format="GTiff", overwrite=TRUE)

#### /!\ projection system for the prediction and sd raster are in EckertIV which is not ideal for Mongolia

#### For assignment we changed the projection to Lambert Conformal Conic (std parallel 1: 40, std parallel 2: 52)

library(raster)

pred<-raster("D:/boulot/mobisteppe/burgast_archeo/manuscrit/soumission/sr_isoscape_pred.tif")

pred<-raster("Output\\sr_isocape_pred.tif")

error<-raster("Output\\sr_isocape_pred.tif")

crs_lcc <- "+proj=lcc +lat_1=40 +lat_2=52 +lat_0=46 +lon_0=104 +x_0=2000000 +y_0=0 +datum=WGS84 +units=m +no_defs"

pred_lcc <- projectRaster(pred, crs = crs_lcc)

error_lcc <- projectRaster(error, crs = crs_lcc)

writeRaster(pred_lcc, filename="Output\\sr_isocape_pred_LCC.tif", format="GTiff", overwrite=TRUE)

writeRaster(error_lcc, filename="Output\\sr_isocape_sd_LCC.tif", format="GTiff", overwrite=TRUE)

writeRaster(pred_lcc, filename="D:/boulot/mobisteppe/burgast_archeo/manuscrit/soumission/sr_isoscape_pred_LCC2.tif", format="GTiff", overwrite=TRUE)

################################################################################

######################## Geographic assignment ################################

################################################################################

library(assignR)

library(raster)

library(zoo)

library(plotrix)

library(car)

library(plotfunctions)

library(terra)

library(readxl)

tabsr <- readxl::read_excel("SM8_Table_S9_horse_isotope_data.xlsx",col_names=TRUE, na="NA", sheet="87Sr86Sr_data")

tabsr <- as.data.frame(tabsr)

lvind<-unique(tabsr$indiv)

pred<-rast("D:/work/isoscape/output/sr_isoscape_pred_LCC.tif")

sdev<-rast("D:/work/isoscape/output/sr_isoscape_sd_LCC.tif")

####select scale for the assignment area

#ex<-ext(-1050000,-950000,460000,560000) #50km

#ex<-ext(-1100000,-900000,410000,610000) #100km

ex<-ext(-1200000,-800000,310000,710000) #200km

rasbrick<-c(pred,sdev)

names(rasbrick)<-c("pred","sd")

rasbrick_cut<-crop(rasbrick,ex)

folder1<-"_burgast_200km" #select 50km, 100km, 200km according to the scale

dir.create(paste("D:/work/assignement/",folder1,sep=""))

for (i in 1:length(lvind))

{

tabi<-subset(tabsr,tabsr[,1]==lvind[i])

lvteeth<-unique(tabi$teeth)

folder2<-lvind[i]

dir.create(paste("D:/work/assignement/",folder1,"/",folder2,sep=""))

for (j in 1:length(lvteeth))

{

print(paste(lvind[i]," - ",lvteeth[j],sep=""))

folder3<-lvteeth[j]

dir.create(paste("D:/work/assignement/",folder1,"/",folder2,"/",folder3,sep=""))

dir.create(paste("D:/work/assignement/",folder1,"/",folder2,"/",folder3,"/rast_raw",sep=""))

dir.create(paste("D:/work/assignement/",folder1,"/",folder2,"/",folder3,"/rast_qtla",sep=""))

#compute average Sr with moving window

ind<-subset(tabi,tabi$teeth==lvteeth[j])

ind<-ind[!is.na(ind$Sr),]

serind<-zoo(ind$Sr,ind$dist_from_apex2)

wind<-35

mean.sr<-rollmean(serind,wind,align="center")

sd.sr<-rollapply(serind,wind,sd,align="center")

serALLmean<-as.vector(mean.sr)

serALLsd<-as.vector(sd.sr)

samp<-index(mean.sr)

indSr<-as.data.frame(cbind(samp,serALLmean,serALLsd))

names(indSr)<-c("measurement","value","sd")

indSr2<-indSr[seq(1,nrow(indSr),10),] #take one sample every 10 samples

dataSr<-indSr2[,c(1,2)]

#spatial assignment

asn<-pdRaster(rasbrick_cut,unknown=dataSr)

qtla<-qtlRaster(asn,threshold=0.1,thresholdType="area")

print(paste("assignment done: ",Sys.time(),sep=""))

####raster raw: raster of raw probability

for (k in 1:dim(asn)[3])

{

ras_asn<-asn[[k]]

ras_norm<-ras_asn

# ras_norm<-ras_asn/max(na.omit(values(ras_asn))) #to normalize the probability of origin if needed

writeRaster(ras_norm,paste("D:/work/assignement/",folder1,"/",folder2,"/",folder3,"/rast_raw/",lvind[i],"_",lvteeth[j],"_",sprintf("%05d",ind[10*(k-1)+18,3]),".tif",sep=""))

}

####raster qtla: raster with 10% of the study area with the highest probability of origin

for (k in 1:dim(asn)[3])

{

ras_qtla<-qtla[[k]]

# ras_norm<-ras_asn/max(na.omit(values(ras_asn))) #to normalize the probability of origin if needed

writeRaster(ras_qtla,paste("D:/work/assignement/",folder1,"/",folder2,"/",folder3,"/rast_qtla/",lvind[i],"_",lvteeth[j],"_",sprintf("%05d",ind[10*(k-1)+18,3]),".tif",sep=""))

}

print(paste("rasters saved: ",Sys.time(),sep=""))

}

}
