# Supplementary material for: 3000 yr-old patterns of mobile pastoralism revealed by multiple isotopes and radiocarbon dating of ancient horses from the Mongolian Altai
Source: PLoS One. 2025 May 7;20(5):e0322431. doi: 10.1371/journal.pone.0322431 (PMC12057990; doi:10.1371/journal.pone.0322431)
Supplement: Attachment — Submitted filename: Response to Reviewers.docx [file pone.0322431.s008.docx]

**Please find our point by point responses (in blue) to the questions and remarks of the reviewers and editor. Line numbers correspond to the track change version.**

The manuscript is overall well-written and the work of interest. I concur with reviewers that you should propose alternative explanations for the non-local signatures of some of the horses and/or in general tone down your interpretations.

Here a few minor edits from myself:

Line 261: please use laser energy (fluence J/cm^2) rather than a %.

We changed the % to the fluence value.

Line 267: the mean value of nist1400 has 5 decimals while the error has 6.

We apologize, the NIST1400 provided here was wrong, as it had been used as a placeholder in an older version of the manuscript. We have corrected the value with the real measurements and have reworked this section to add additional information.

Line 286: As disclosed by Bataille and coauthors in their own 2020 paper on the global isoscape, using the whole global dataset for predicting an under-represented area can decrease the accuracy of the prediction itself (see the Madagascar example). I’m not saying you should repeat the whole analysis, yet you should likely test the performance of the RF model with the Altai data only.

Using only Altai data to run the RF model, we obtained a model with a slightly better RMSE (0.0022 instead of 0.0035) but that explain only 37.1% of the variance (61.6% with the full dataset). We added the following sentence line 396:

"Running the model of Altai data only lead to a slightly improved RMSE (0.0022) but with a lower explanatory power of 37.1% of the variance.”

Line 422: This sentence can be interpreted as environmental changes of the Sr (as observed e.g. in tropical areas) rather than a seasonal mobility of the horse.

We changed the sentence to : “This similarity between the two signals suggests that changes in ^87^Sr/^86^Sr values are driven by seasonal movements”

5. Review Comments to the Author

Please use the space provided to explain your answers to the questions above. You may also include additional comments for the author, including concerns about dual publication, research ethics, or publication ethics. (Please upload your review as an attachment if it exceeds 20,000 characters)

Reviewer #1: I believe that Zazzo et al.’s paper represents a good example of multi-isotope analysis for exploring animal mobility in the context of Western Mongolia and a valuable contribution for mobility studies in the area, as it presents an updated regional isoscape of bioavailable Sr. The methodology is sound (I very much appreciated the use of modern horse as a check-test), results are consistent, and references updated. Therefore, I recommend the publication of the manuscript on PLOS ONE, although I suggest to address some relevant issues regarding the general structure of the paper and the final interpretation of the isotopic results, as regards to the relationship with the archaeological context.

First, to improve the clarity of the main text, I think that there are too many references to the supplementary information. As PLOS ONE has no page limit, perhaps some of the figures that are currently in the SI, might be put in the main text. FigS1, FigS2, for example, are extremely important for the interpretation of the context. I found the presentation of the isotopic results a bit hard to read. Instead of subdividing paragraphs by isotopes, why not by horse? As only 6 horses are analysed in such a detailed resolution, it would be great to present each single “life history”, and only at the end, a synthesis of all data.

We moved the figures S1 and S2 to the main text and adjusted the figure numbers in the main text and in the SI accordingly.

We kept the sections about δ^18^O and δ^13^C as they were, as there were no distinct types of profiles among the individuals. However, we reworked the ^87^Sr/^86^Sr and assignment section. This section is now divided in three sub-sections: local horse displaying seasonal mobility, local horse with no apparent mobility, non-local horse. For a given archetype, intra-tooth ^87^Sr/^86^Sr profiles are presented with the corresponding geographic assignment to avoid repetition.

My second point concerns the archaeological contexts. Can we exclude that the horses were sacrificed for celebrating the deceased’s death? I read at lines 602-603 that “previous work by our team showed that not all horses in a khirgisuur died at the same time of the year”. Looking at Lazzerini et al.’s paper (2020), only two horses were analysed, ST 61 and 66. The skulls ST63, 64, 65, 66 might therefore be simultaneous (also judging from the planimetry). The pits in which skulls were deposed, in fact, seem well connected to the burial (FigS1). In this case, a single event, at least for ST63, 64, 65, 66 cannot be ruled out, as far as I understood. This would be corroborated by the homogeneity of radiocarbon dates (0-104 y, see line 356), with the obvious limitations derived from the 14C resolution. Eventually stratigraphic evidence can help in addressing the issue of synchrony /diachrony. For example, ST63, 64, 65, 66 structures are very close to each other. Do some of the pits cut other pits (which would indicate a sequence of events delayed in time)? Moreover, the deposition of animal skulls, at least to my opinion, seems more typical of ritual performed in funerary areas, as documented in various Bronze and Iron Age contexts across Eurasia.

This is a very good point indeed. We confirm that we cannot rule out a single event for horses 63-66. For Burgast, see Lepetz et al., 2022. For larger khirgisuurs horses, the deposits span about fifty years (Zazzo et al., 2019). These issues have been debated in Lepetz et al., 2019 and Zazzo et al., 2019 , as well as Lazzerini et al., 2020. Furthermore, it is not possible to establish a relative chronology between the different deposits, as the pits do not crosscut each other. However, the concept of a single event is relative. The horse heads may have been deposited over the course of a single day, week, month, or several months. It is impossible to assess the frequency or rhythm of these deposits. What is certain however, is that the horses did not all die at the same time of year, thus excluding a single sacrifice sequence at the burial site. We have added a few lines in the introduction (line 78-80 and 83-90) to better explain this, but also refer to the published articles by our team.

At line 157-161 the Authors state that “*On the eastern side, there are seven mounds (ST 61 to 67) yielding horse remains of 7 individuals, mainly represented by their skulls or mandibles and sometimes accompanied by connected cervical vertebrae and terminal phalanges. The horse remains are fairly well preserved, but bone displacements, breakage and missing parts seem to indicate that taphonomic disturbances (possibly by burrowing animals) have occurred, although it is not always possible to precisely define their extent”.*

As expert of human and animal bones, I can say that it is quite a unique case in which cervical vertebrae and phalanges are preserved, and long bones diaphysis are not. Trabecular bone is much more fragile than cortical bone. Therefore, isn’t it possible that the skulls (+ cervical vertebrae, etc.) were the only parts that were deposed? And why only cervical vertebrae and not thoracic/lumbar/sacral/caudal vertebrae? Is it a coincidence, or the horses were beheaded and sacrificed? Moreover, what is the size of the deposition pits (it is not very clear from Fig1)? Are they large enough to contain the whole body of a horse (they are not, judging from Lazzerini et al. 2020, Fig. 2, which would be much better than Fig.1 of the manuscript)? This is just a hypothesis but, looking at the way the evidence is presented in the manuscript, the most convincing, at least for individuals 63-66. If the authors have archaeological or archaeozoological data to exclude it, I suggest addressing the issue in the main text.

Only the skulls, sometimes neck vertebrae and terminal phalanges, have been deposited. We have added a few sentences in the introduction section (line 78-80 and 83-90) for readers unfamiliar with archaeological sites in Mongolia. Further discussion of these issues here does not seem necessary, as they are addressed in the cited articles.

Following this line, in case the horses were sacrificed, for example, should we consider other alternative ideas, for example that the horses were spoils of war (taken from different enemies)? Celebration of victory in conflict? Gifts to a war chief? Sacrificial victims for accessing the afterlife? In that case, their mobility (or the mobility of some of them) could be related to different purposes that simply grazing. What I am emphasizing, for the benefit of the authors, is that the thesis they present assumes that the deposition mirrors simply herds, or different herds. I do not disagree. However, the presentation of the archaeological context should be more detailed, and the final conclusion should, at least in my opinion, taking into account different (and more complex) alternative options.

Again, we hope that the lines added in the introduction (line 83-90, see above) will help the reader unfamiliar with mongol bronze age archaeology to better understand the arachaeological contexts.

Minor comments:

I see a slight, partial contradiction between the straightforward statement at lines 67-69 “*Mounted nomadic pastoralism was adopted in Mongolia at the beginning of the Late Bronze Age (c. 1200 BCE) and played a significant role in shaping the social, economic and cultural landscape of the region*” and the statement in the conclusion at lines 656-658 “*Although anthropological study can help us keeping in mind the variety of possible circulation modalities, archaeology can, however, not document the intricacies of exchange practices for the contexts of late Bronze Age Mongolia*”.

Either we have a clear picture of LBA pastoralism in Mongolia, or archaeological evidence of animal circulation is too scarce/not deeply investigated. Eventually instead of just mentioning Honeychurch’s works, it would be clearer for the readers what the LBA archaeological evidence tell us about this significant role.

We are not sure to understand the contradiction here. It may be due to a misunderstanding of the therm « circulation ». By circulation we mean exchange of animals between herding family, not seasonal movements of animals (which defines nomadic pastoralism). The term circulation includes (but is not limited to) exchange. For example, gift of theft is not what we can call an exchange, but leads to a circulation of animals. This term ‘circulation’ is well defined in the preceding sentence : « Animal circulation between herders, which can take the form of exchange, trade, gift or theft, are well documented in social anthropology (e.g. [81,82] for African pastoralists)». For this reason, we feel that there is no contradiction in the two sentences mentionned by the reviewer.

647: in which sense “contemporary nomadic lifestyles […] reflect responses to socio-economic conditions”? Does it mean that herder change routes depending on the economic resources they have or that herders having different economic statuses use different routes/pastures?

To clarify, we rephrased the sentence below (now line 781)

« The flexibility of contemporary nomadic lifestyles allows communities to respond to environmental changes and mitigate risks associated with climate variability. They also reflect responses to shifting socio-economic conditions. While the nomadic itineraries of herders are typically cyclical, variations in seasonal mobility practices have also been documented on a smaller geographic (local) and temporal (interannual) scale in Mongolia (e.g. in Bayan-Ölgii Province [42,71,78], and in Arkhangai Province [70,79,80]). »

as follows :

« The flexibility of contemporary nomadic lifestyles allows communities to respond to changing conditions, which particularly allows them to mitigate risks associated with climate variability for example. While the nomadic itineraries of herders are typically cyclical, variations in seasonal mobility practices, whether for socio-economic or environmental reasons, are thus common and have also been locally documented in Mongolia, including on an interannual scale (e.g. in Bayan-Ölgii Province [42,71,78], and in Arkhangai Province [70,79,80]). »

658: Late Bronze Age Mongolia

Reviewer #2: The article by Zazzo et al regarding mobile pastoralism in Mongolia is very well done on the scientific side. Their use of combined Sr, O, and C isotopes is well thought out and the results are promising. I agree with the majority of their findings, but they need to be stated more strongly in the discussion and conclusion. About midway through the paper the text becomes a bit repetitive and needs some editing. It might be that some of the detail of isoscape construction, etc, could be moved to the supplement.

We simplified the random forest section and moved details into the supplementary material SM4.

One issue with the paper is the consistent use of the term nomadic, even when they are proposing to study mobility. This is seen in the introduction, line 66 when they discuss the adoption of ‘mounted nomadic pastoralism’ being adopted. It remains unclear why they did not mention mounted pastoralism, as the ‘nomadic’ part is the question under study. Were early horse riders nomadic, or not?

This happens again on line 90, where they say that they want to address the study of pastoral nomadic mobility – it is enough to say pastoral mobility. The word nomadic implies that you already know the answer to the question.

Part of the issue here is that the word « mobility » remains quite vague and includes ‘daily’ as well as ‘seasonal’ mobility. Therefore we now stress in the paper that the mobility we are referring to is seasonal and replace (on line 47 for example) nomadic by « pastoral » as suggested. Below are the other places where the term was edited/removed :

lignes 68 and 138 : we removed the term nomadic

ligne 95 we replaced « nomadic » by « seasonal »

ligne 694 we remplaced « nomadic routes » by « mobility patterns »

ligne 778 : we remplaced « nomadic territory » par « grazing areas » which is more neutral (and because we record what was grazed by the horse anyways).

Starting at line 211, there are some issues in this section. Line 217, ‘changes in the relative abundance of WILD C3 and C4 plants’ – but there should be a caveat here that the carbon isotope values of enamel could also support evidence for foddering with wild or domesticated plants – which is part of the reason the carbon isotopes of enamel cannot be used as a proxy for elevation – because humans are managing horses.

Today, foddering is limited in this region, males being foddered only during harsh winters while females and foals can but not systematically receive fodder for a limited period of time (Lazzerini et al. 2021). Controlled feeding studies also suggest that short supplementation has a limited impact on δ^13^C intra-tooth signal (e.g. Zazzo et al. 2010). We therefore believe that the effect of foddering on enamel d13C values is marginal. We developed this point in the discussion line 656:

Our experience shows that in modern practices, males are only (lightly) supplemented during harsh years, while females and foals only receive fodder in winter for a limited period of time [43]. Although effect of foddering cannot be entirely ruled out, it is likely marginal, as short-term supplementation in C_4_ plants should not cause a significant increase in δ^13^C values [78].

I am not sure that the Bayesian geographic assignment was necessary, but if you are going to use this then please alter the figures so that it is clear that the colors are elevation rather than isotope values. It is pretty confusing.

A Bayesian approach is optimal for spatial assignment on continuous-probabilistic surfaces such as ^87^Sr/^86^Sr isoscapes generated from a Random Forest because it naturally incorporates the probabilistic nature of the model and the associated uncertainties. This method improves accuracy by weighting geographic regions based on likelihood, providing a more robust and nuanced assignment than deterministic approaches. We added this sentence line 359 to justify the use of our approach:

“The Bayesian approach is particularly suited for geographic assignment on continuous surfaces such as the isoscape generated with the RF, accounting for the probabilistic nature of the model and the associated uncertainties.”

The assignment maps depict the most likely areas of origin of a given sample with the 10% of the surface of the map with the highest probability of origin. Given the importance of altitudinal movements in modern pastoralism in this region of Mongolia, we kept the elevation representation in brown color scale, as indicated below the map, distinct from the ⁸⁷Sr/⁸⁶Sr and isoscape uncertainty color scales in Fig. 8. Although we placed less emphasis on δ¹³C and elevation based on the reviewer’s comments, altitudinal movements remain relevant. Therefore, we decided to maintain the maps in their original form. However, we clarified the legend of the figures and provided a better explanation of the figures in the results line 462:

“For a given sample, the top 10% most probable origin areas are displayed on the map with elevation overlaid within these areas for interpretation, as altitudinal movements are a key aspect of pastoralism in this region of Mongolia.”

Finally, in response to the reviewer’s comment on image quality, we have increased the resolution and verified it using PLOS ONE's tools to ensure better readability.

Lines 393 through 404, this description of correlations between carbon isotopes of tooth enamel and elevation is spurious. It does not help with your argument and actually incorporates confusion into your argument. I would cut this and any discussion of possible correlations between d13C of enamel and predictions of elevation.

The relationship between δ¹³C and elevation is based on the assumption that plant communities, particularly the relative abundance of C₃ versus C₄ plants, increase with elevation due to environmental variations along the altitudinal gradient. We provided additional references in the method to support this assumption. This should translate by a decrease in the mean δ¹³C of the diet as C_3_ plants have a lower δ¹³C value than C_4_ plants. The equation we used was specifically developed for modern horses in the Altai (Lazzerini et al. 2021), along with a similar equation for caprines. According to this study, individuals that spent a long period of time at high elevation presented averaged δ¹³C values significantly lower than individuals that made short incursion at high elevation.

However, we acknowledge that the relationship between δ¹³C and elevation is weak, and variations in δ¹³C along teeth are likely not solely attributable to changes in altitude. Moreover, the fact that our predicted elevations do not align with observed elevations in the Altai Mountain range—even for the modern horse—raises questions about the reliability of δ¹³C as a direct proxy for elevation. That said, variations in δ¹³C, particularly the negative covariation between δ¹⁸O and δ¹³C observed in several individuals, remain noteworthy and may indicate movements across different altitudes.

We have revised the δ¹³C section line 437-444, replacing *“elevation used by”* with *“elevation predicted”* and down toned our interpretation of δ¹³C data (line 447-9). Additionally, we removed the use of δ¹³C profiles to refine ⁸⁷Sr/⁸⁶Sr assignments in the spatial assignment section. Finally, we added a paragraph at line 651-60 discussing the limitations of using δ¹³C to infer elevation.

Many of the figures, especially 4 and 5, are very blurry and unreadable. Please remake or increase the size.

We were surprised by this comment and apologize for the inconvenience. Our figures meet high-quality standards, exceeding the dimensions recommended by *PLOS ONE* and maintaining a resolution of 300 dpi. We did not encounter any issues during our own check of PDF conversion. However, we have revised the figures, increasing the resolution to 600 dpi, and verified their quality using *PLOS ONE*’s validation tool to ensure better readability.

The geographic assignments are ok, but the maps are unclear. The yellow and red (supposed to be elevation?) do not help the reader. It would be better if these were given colors that correspond to strontium isotope values so that we can see the various locations where horses may have spent time.

As mentioned above, these maps aim to highlight the most likely areas of origin and their elevation, given the importance of altitudinal movements in pastoralism in this region. Each assignment map corresponds to a specific ⁸⁷Sr/⁸⁶Sr value taken along the intra-tooth profile, identifying the most likely areas of origin for that value. Thus, displaying ^87^Sr/^86^Sr value within the assigned area would result in a largely uniform color reflecting the sample’s ^87^Sr/^86^Sr value. While this color may vary between maps, it remains consistent within each individual map. Given these considerations, we strongly believe that keeping the maps in their current form is the most appropriate way to convey the data effectively.

Smaller detailed edits:
Line 76 should read ‘Some khirigsuurs are quite small containing fewer than ten horse head mounds, while others can reach hundreds of horse head mounds’

That is correct. Correction done.
Line 153 should say ‘…with most khirigsuurs, the main burial is associated….’

Correction done.
Line 345 the word Given should be lowercase

Correction done.
Lines 368 to 369, the authors need to explain the RMSE and why they think the Rsquared value of 0.67 is important.

We added an explanation of RMSE at its first appearance (Line 397):

“The RF model demonstrates a root mean square error (RMSE) of 0.0035, which quantifies the mean deviation between predicted and actual values, and an explanatory power of 61.6% of the variance.”

We also clarified why RMSE and R² were assessed for Sr observed in plants versus Sr predicted by the RF (Line 409):

“The ^87^Sr/^86^Sr values from the plants sampled in Altai demonstrate a good agreement with the ^87^Sr/^86^Sr values from the isoscape at the sampling sites (RMSE = 0.0015, R² = 0.67), indicating that ^87^Sr/^86^Sr predicted by the RF is reliable in this region.”

Line 390, what is a solution analysis in this context?

We changed "solution" to "IRMS".

Line 392, this should say ‘…and predicted altitude’

Everything is predicted (mobility, seasonality, altitude). So we kept it as is for convenience.

Line 500 should say ‘…historical pastoral systems’

ok
Line 506 remove nomadic from these discussions, it should say mobility practices. Mountain nomadism should be vertical mobility

we are not sure to understand this comment. We are referring here to the work of 20^th^ century geographers who have studied modern nomads, so there is no reason to doubt that they were nomads.

Lines 519 to 523 – it seems like the spatial error is significant. How can spatial error of >0.001 not be problematic?

The Bayesian assignment approach explicitly integrates uncertainty by identifying the most probable areas based on probability distributions rather than absolute values. Even with high uncertainty, geographic signals remain detectable. When a sample has a Sr value that falls within the isoscape’s range, it will be assigned to a broader area due to the uncertainty, but the relative probability ranking of the most likely regions will remain unchanged. However, if a sample's Sr value falls outside the isoscape’s range, the assignment is more likely to favor areas with high uncertainty, as these regions allow for a greater probability of observing extreme values. This is why we do not infer a specific area of origin for the low Sr values of horse ST62 and instead limit our interpretation to a non-local origin relative to the Sr baseline of the Altai region.

We added explanations in the “bioavailable isoscape and geographic assignments” section (line 649-54):

“We used a bayesian assignment approach to take into account the relatively high spatial uncertainty associated with the RF prediction. The Bayesian assignment explicitly integrates uncertainty by identifying the most probable areas based on probability distributions rather than absolute values [ref wunder]. Even with high uncertainty, geographic signals remain detectable. Assignment areas are broadened but the relative probability ranking of the most likely regions will remain unchanged.”

And more specifically for horse ST62 line 765:

“However, these areas must be treated with caution, as the low ^87^sr/^86^Sr values of ST62 fall out of the isoscape range, meaning the assignment of these samples may be biased toward high-uncertainty areas where extreme values are more probable.”

Line 603 – remove ‘his horses’, maybe their horses?

Ok (now 742)

Lines 644, 645 – remove nomadic when discussing mobility practices.

As we are referring here to 20^th^ century nomads, there is no reason to remove the term nomads. So we kept it as is.
